# Suppression of endogenous retroviral enhancers in mouse embryos derived from somatic cell nuclear transfer

**DOI:** 10.3389/fgene.2022.1032760

**Published:** 2022-11-08

**Authors:** Daiki Shikata, Shogo Matoba, Masashi Hada, Akihiko Sakashita, Kimiko Inoue, Atsuo Ogura

**Affiliations:** ^1^ Bioresource Engineering Division, RIKEN BioResource Research Center, Tsukuba, Ibaraki, Japan; ^2^ Graduate School of Life and Environmental Sciences, University of Tsukuba, Tsukuba, Ibaraki, Japan; ^3^ Cooperative Division of Veterinary Sciences, Tokyo University of Agriculture and Technology, Fuchu, Tokyo, Japan; ^4^ Laboratory of Pathology and Development, Institute for Quantitative Biosciences, The University of Tokyo, Bunkyo-ku, Tokyo, Japan; ^5^ Department of Molecular Biology, Keio University School of Medicine, Shinjuku-ku, Tokyo, Japan; ^6^ RIKEN Cluster for Pioneering Research, Wako, Saitama, Japan

**Keywords:** somatic cell nuclear transfer, endogenous retrovirus, epigenetics, enhancer, RNA sequencing

## Abstract

Endogenous retroviruses (ERVs) in the mammalian genome play diverse roles in embryonic development. These developmentally related ERVs are generally repressed in somatic cells and therefore are likely repressed in embryos derived from somatic cell nuclear transfer (SCNT). In this study, we sought to identify ERVs that are repressed in SCNT-derived morulae, which might cause previously unexplained embryonic deaths shortly after implantation. Our transcriptome analysis revealed that, amongst ERV families, ERVK was specifically, and strongly downregulated in SCNT-derived embryos while other transposable elements including LINE and ERVL were unchanged. Among the subfamilies of ERVK, RLTR45-int was most repressed in SCNT-derived embryos despite its highest expression in control fertilized embryos. Interestingly, the nearby genes (within 5–50 kb, *n* = 18; 50–200 kb, *n* = 63) of the repressed RLTR45-int loci were also repressed in SCNT-derived embryos, with a significant correlation between them. Furthermore, lysine H3K27 acetylation was enriched around the RLTR45-int loci. These findings indicate that RLTR45-int elements function as enhancers of nearby genes. Indeed, deletion of two sequential RLTR45-int loci on chromosome 4 or 18 resulted in downregulations of nearby genes at the morula stage. We also found that RLTR45-int loci, especially SCNT-low, enhancer-like loci, were strongly enriched with H3K9me3, a repressive histone mark. Importantly, these H3K9me3-enriched regions were not activated by overexpression of H3K9me3 demethylase Kdm4d in SCNT-derived embryos, suggesting the presence of another epigenetic barrier repressing their expressions and enhancer activities in SCNT embryos. Thus, we identified ERVK subfamily RLTR45-int, putative enhancer elements, as a strong reprogramming barrier for SCNT (253 words).

## Introduction

Somatic cell nuclear transfer (SCNT) in mammals is the sole reproductive engineering technique that produces new individuals from single somatic cell nuclei acting as genome donors. Besides its promising applications in stock animal industries, regenerative medicine and biopharmaceutical production, SCNT can also provide unique research models for the study of developmental epigenetics (Simmet et al., 2020; Ogura et al., 2021). Epigenetic or transcriptomic analyses of SCNT-derived embryos, fetuses and placentas are expected to identify the molecular mechanisms of genomic reprogramming as well as those related to epigenomic dynamics during the life cycle in mammals. In particular, mouse SCNT has greatly contributed to the field because of the availability of abundant genetic information and genetically defined strains, which ensure reproducibility and reliability of the research results (Matoba and Zhang, 2018; Ogura et al., 2021).

The birth of cloned mice was first reported in 1998, as the second cloned mammalian species reported in that year, following cloned bovines. However, the birth rates of mice cloned by SCNT were generally lower than those of farm animals, ranging from 1% to 3% per embryos transferred (Wakayama et al., 1998). Since then, many researchers have attempted to improve mouse cloning *via* SCNT, based on the ideas of ameliorating epigenetic abnormalities associated with the procedure. The first significant step was the treatment of one-cell SCNT-derived embryos with trichostatin A, a potent histone deacetylase inhibitor, expecting an increase in the accessibility of the ooplasmic factors to nucleosomes of the donor genome (Kishigami et al., 2006; Rybouchkin et al., 2006) and thus an acceleration of zygotic gene activation (Inoue et al., 2015; Kamimura et al., 2021; Yang et al., 2021). The second successful improvement of mouse SCNT was achieved by normalizing the expression pattern of the *Xist* gene, a noncanonical imprinted gene responsible for X chromosome inactivation (Inoue et al., 2010; Matoba et al., 2011). Because the imprinting memory of *Xist* has been erased in somatic cells including trophoblasts (Oikawa et al., 2014; Inoue et al., 2017), *Xist* is ectopically expressed in SCNT-derived preimplantation embryos, leading to a large-scale suppression of X-linked genes in such embryos. The third significant step for the improvement of mouse SCNT was activation of the donor cell-derived H3K9me3-enriched regions containing genes specifically repressed in SCNT-derived embryos (termed “reprogramming resistant regions”, RRRs) by a specific histone demethylase, Kdm4d (Matoba et al., 2014; Hada et al., 2022). More recently, we and others have confirmed that loss of H3K27me3-dependent imprinting (noncanonical imprinting) inherited from the donor somatic cells was the major cause of SCNT-specific placental enlargement (Matoba et al., 2018; Inoue et al., 2020; Wang et al., 2020; Xie et al., 2022). Other epigenetic marks such as DNA methylation and the H3K27me3 mark have also been modified to improve mouse SCNT (Gao et al., 2018; Yang et al., 2018; Zhou et al., 2019).

Thus, the efficiency of mouse SCNT has been improved consistently by ameliorating the epigenetic abnormalities associated with the technology. It should be noted that specific aberrations in epigenetic patterns can be found in preimplantation embryos, preceding SCNT-associated abnormal phenotypes such as death of embryos during the postimplantation period (Matoba et al., 2011; Gao et al., 2018). Indeed, a recent study demonstrated that transient *Xist* upregulation, induced by loss of maternal polycomb PRC2, in the preimplantation period could show a detrimental ripple effect resulting in postimplantation death in mice (Matoba et al., 2022). Therefore, it is important to identify other SCNT-specific changes in the epigenome or gene expression patterns during preimplantation development for further technical improvements of SCNT technology.

It is known that preimplantation mammalian embryos activate a number of development-related embryonic genes, which include retrotransposons and endogenous retroviruses (ERVs). For example, MERVL, a typical ERV-derived element in the mouse, is transcribed during major zygotic gene activation (ZGA) (Kigami et al., 2003). Furthermore, abundant chimeric transcripts containing such elements were also identified as putative drivers of mouse ZGA (Peaston et al., 2004; Macfarlan et al., 2012). ERVs are preferentially localized in gene-poor regions, which are largely enriched with a repressive histone mark, H3K9me3, in somatic cells (Kato et al., 2018). Importantly, MERVL elements are included in the abovementioned RRRs, H3K9me3-enriched regions (Matoba et al., 2014), and the MERVL elements were re-activated following injection of mRNA for the H3K9me demethylase Kdm4d, contributing to improved developmental efficiency of SCNT-derived embryos (Matoba et al., 2014).

This scenario prompted us to search for other sets of ERVs that are specifically repressed in SCNT-derived embryos. We hypothesized that these sets might be developmentally important because they are known to be capable of regulating nearby genes by providing alternative promoters or enhancers (Chuong et al., 2013; Todd et al., 2019; Modzelewski et al., 2021). ERVs that are essential for early embryonic development might be repressed in somatic cells because transposable elements (TEs) in general may be harmful to the genomic integrity when they are ectopically expressed. We then focused on the analysis of morula-stage embryos, expecting that we could detect the transcriptional dysregulation of ERVs that might affect postimplantation development of SCNT-derived embryos. Interestingly, we identified subsets of enhancer-like endogenous retroviruses that were specifically downregulated in SCNT-derived morulae. Our data provide new clues to define the epigenomic characteristics of SCNT-derived embryos that might be the cause of their poor developmental potency after implantation.

## Results

### Comparative transcriptomic analysis of *in vitro* fertilization- and somatic cell nuclear transfer-derived morulae

**FIGURE 1:**
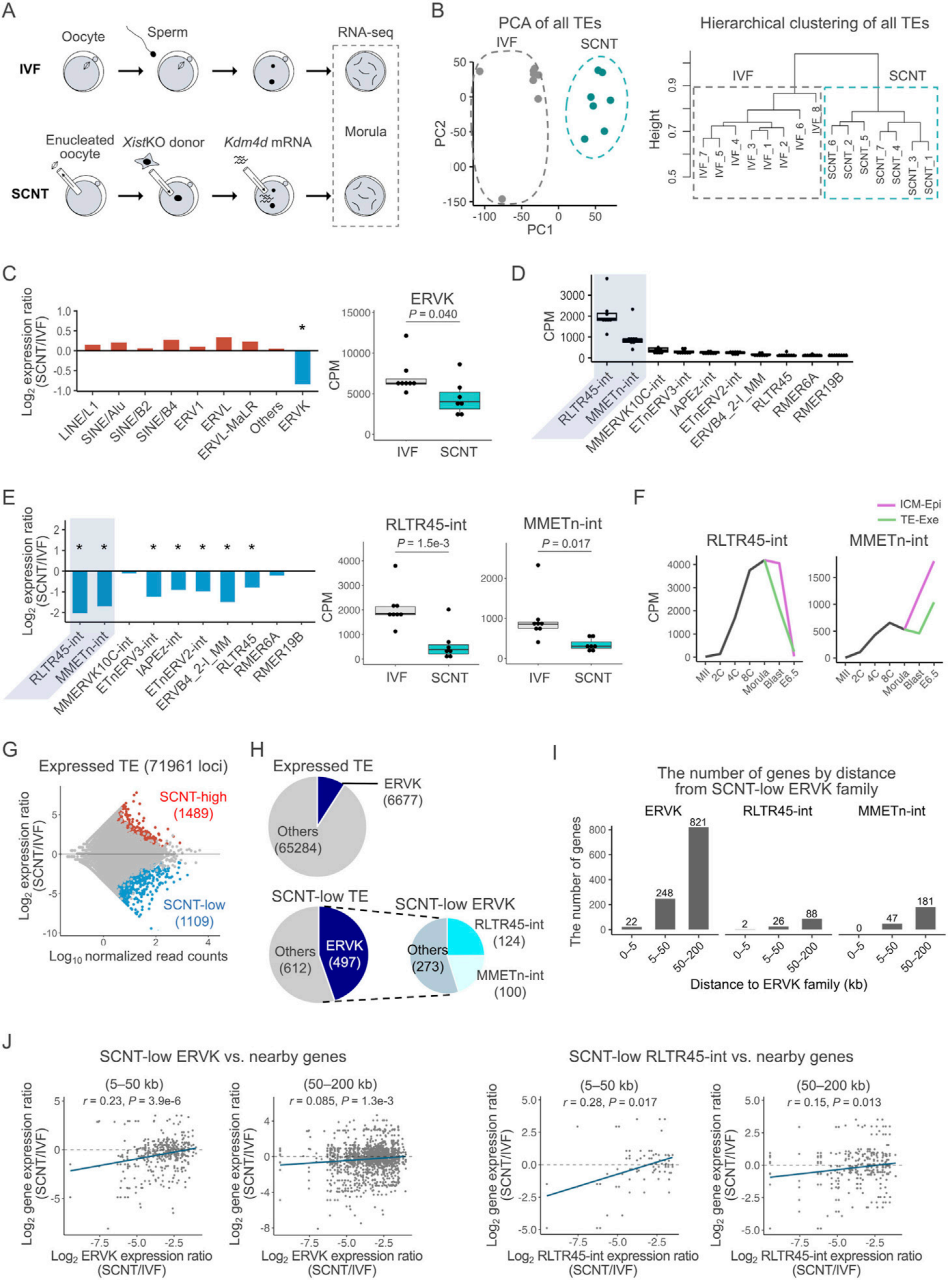
Expression of ERVK was suppressed in SCNT-derived embryos. **(A)** Schematics of the experimental design. Embryos at the morula stage were used for single-embryo RNA-seq analysis **(B)** PCA of the genome-wide TE expressions in IVF and SCNT-derived embryos. Grey and blue dots represent IVF- and SCNT-derived embryos, respectively (left). Hierarchical clustering analysis of the genome-wide TE expressions in IVF- and SCNT-derived embryos (right). **(C)** Bar graph showing the log_2_ expression ratio of each TE family in IVF and SCNT-derived embryos (left). Box-and-whisker plots comparing the expression of ERVK between IVF- and SCNT-derived embryos. *P* was calculated by a two-tailed *t*-test (right). *p < 0.05. **(D)**Box-and-whisker plots showing the expressions of the top 10 most highly expressed ERVK subfamilies. RLTR45-int and MMETn-int are highlighted in gray **(E)** Bar graph showing the log_2_ expression ratio of each ERVK subfamily in IVF and SCNT-derived embryos (left). Box-and-whisker plots comparing the expression of RLTR45-int and MMETn-int between IVF- and SCNT-derived embryos. *p* values were calculated by a two-tailed *t*-test (right). *p < 0.05. **(F)** Line graph showing the average expression levels of RLTR45-int and MMETn-int throughout early embryonic development. Published RNA-seq data (GSE98150) were analyzed. The samples used in the analysis are MII oocytes (MII), 2-cell- (2C), 4-cell- (4C), 8-cell- (8C), and morula-stage embryos, inner cell mass (ICM) and trophectoderm (TE) of blastocysts, and epiblast (Epi) and extraembryonic ectoderm (Exe) of E6.5 embryos. **(G)** MA plot showing the TE expression ratio between SCNT- and IVF-derived embryos. Among 71,961 expressed TE loci, 1,489 were significantly upregulated (red circles) and 1,109 were downregulated (blue circles) in SCNT-derived embryos. Expressed TE loci were defined as those with at least one read on average per embryo. Differentially expressed TE loci were defined as those with absolute log_2_ expression ratio ≥1 and an adjusted *p*-value < 0.1 using DESeq2. **(H)** The upper pie chart shows the number of expressed ERVK loci out of the total expressed TE loci. The lower pie charts show the number of SCNT-low ERVK loci out of the total SCNT-low TE loci. **(I)** Bar graph showing the distribution of genes adjacent to SCNT-low ERVK, RLTR45-int, and MMETn-int. **(J)** Scatter plot showing the Spearman correlation between the SCNT/IVF expression ratio of SCNT-low ERVK (left) or RLTR45-int (right) loci and that of their adjacent genes. Adjacent genes are defined as genes with transcription start site located within 5–50 kb or 50–200 kb up/downstream from a ERVK or RLTR45-int locus. The blue line indicates the regression line. *p* values and correlation coefficients were calculated using the “cor” function in R.

In this study, we generated SCNT-derived embryos using *Xist* maternal knockout donor cells in a B6D2F1 background, followed by injection of *Kdm4d* mRNA into the reconstructed 1-cell stage embryos (Figure 1A). We employed this experimental protocol to minimize the known epigenetic abnormalities associated with SCNT, expecting identification of new sets of abnormalities in the resulting embryos. The embryos thus prepared were used for transcriptome analysis at the morula stage, using genetic background-matched (B6D2F1) IVF-derived embryos as the controls. First, to confirm the reliability of our transcriptome data, we examined the gene expression profiles of coding genes in IVF- and SCNT-derived morulae. These two types of embryos were clearly separated from each other on the PC1 (principal component 1) axis by principal component analysis (PCA) (Supplementary Figure S1A). The major upregulated genes in SCNT-derived embryos were common to those identified in *Kdm4d*-injected SCNT-derived blastocysts such as *Parva* and *Derl3* (Matoba et al., 2018) (Supplementary Figure S1B). The high-ranked gene ontology (GO) terms of these upregulated genes were composed of specific and diverse developmental categories such as the HIF-1 signaling pathway, anion transport, and immunological regulation (Supplementary Figure S1C). The *Kdm4d* gene was also categorized as upregulated probably because of a remnant of injected *Kdm4d* mRNA. The major downregulated genes in SCNT-derived embryos included those identified in *Xist* knockout SCNT-derived blastocysts such as the *Xlr* and *Magea* family genes (Inoue et al., 2010) (Supplementary Figure S1B). The high-ranked GO terms of these downregulated genes are largely composed of general development-related categories such as methylation, apoptosis, cell proliferation, embryonic morphogenesis, and cell fate specification (Supplementary Figure S1C). These results might indicate that the genes essential for early embryonic development are strongly repressed in somatic cells and are sometimes unable to be reprogrammed by SCNT (Akter et al., 2021). By contrast, for the upregulated genes, it is reasonable to suppose that the cell-type specific genes could be activated promiscuously by genomic reprogramming given their regulatory, not constitutive, epigenomic pattern formation (Trojer and Reinberg, 2007). This might have caused ectopic upregulations of diverse genes by genomic reprogramming following SCNT. Taken together, we considered that our comparative transcriptome data were consistent and reliable enough for further analysis.

### Among the transposable elements, ERVK was specifically repressed in somatic cell nuclear transfer-derived embryos

We then examined the expression profiles of transposable elements (TEs) including ERVs to evaluate whether they were dysregulated in SCNT-derived morulae. Analysis with PCA and hierarchical clustering for all the genome-wide TE expressions clearly distinguished the SCNT-derived from the IVF-derived embryos, indicating that TEs were also differentially expressed in the former as were coding genes (Figure 1B). We next examined what types of TEs showed dysregulation in their expression patterns in SCNT-derived embryos. Interestingly, among the different TE types including LINE/L1 and SINE/B2, only ERVKs were specifically repressed in these embryos (Figure 1C, Supplementary Figure S2A). ERVKs can be classified into different subfamilies according to their components, each of which show a specific expression pattern in preimplantation embryos (Ge, 2017). We then focused on 10 subfamilies of ERVKs that were highly activated in IVF-derived morulae (Figure 1D) and found that most of them (7/10) were repressed in SCNT-derived embryos (Figure 1E, Supplementary Figure S2B). Among them, two ERVK subfamilies with the highest expressions, RLTR45-int and MMETn-int, showed remarkable downregulation in SCNT-derived embryos (Figure 1E, Supplementary Figure S2B). Consistent patterns were observed when we re-analyzed the previous data from 8-cell SCNT embryos derived from embryonic fibroblast cells and SCNT blastocysts derived from cumulus cells (Zhang et al., 2016, 2020; Matoba et al., 2018) (Supplementary Figure S2C,D). During preimplantation development, RLTR45-int and MMETn-int increased their expression levels until the morula stage, and then downregulated or upregulated their expression after implantation, respectively, indicating that they might play different roles at or after implantation (Figure 1F). MMETn-int was more highly expressed in inner cell mass/epiblast cells than in trophectoderm/extraembryonic cells (Figure 1F). Other ERVK subfamilies also showed differential expression patterns during the preimplantation period, indicating that they might play diverse roles around this time (Supplementary Figure S2E).

When we searched for TEs that were differentially expressed between SCNT- and IVF-derived embryos, 1489 and 1109 among 71,961 TEs (loci) were identified as upregulated (“SCNT-high”) and downregulated (“SCNT-low”) TEs, respectively (Figure 1G). Although ERVKs account for only about 9% (6,677/71,961) of all TEs expressed at the morula stage, they comprised 45% of the SCNT-low TEs (Figure 1H). Importantly, nearly half of the SCNT-low ERVK elements were RLTR45-int or MMETn-int (Figure 1H). These biased downregulation patterns of ERVK and RLTR45-int/MMETn-int were also discernible by SCNT-high or SCNT-low classification within each TE/subfamily type (Supplementary Figure S3A). Taken together, we conclude that ERVK elements, especially RLTR45-int and MMETn-int, are the primary TEs specifically downregulated in SCNT-derived morulae.

Next, we examined the possibility that ERVK elements or RLTR45-int/MMETn-int subfamilies might be regulatory elements of nearby genes (5–50 and 50–200 kb) (Figure 1I). The expression ratios (SCNT/IVF) of ERVK and RLTR45-int, but not MMETn-int, showed significant correlations with those of nearby genes at both distance ranges from these loci (Figure 1J, Supplementary Figure S3B). This suggests that ERVK elements, especially RLTR45-int, might be involved in the regulation of nearby genes and may cause their downregulation in SCNT-derived morulae.

### An ERVK subfamily, RLTR45-int, might act as putative enhancers

**FIGURE 2:**
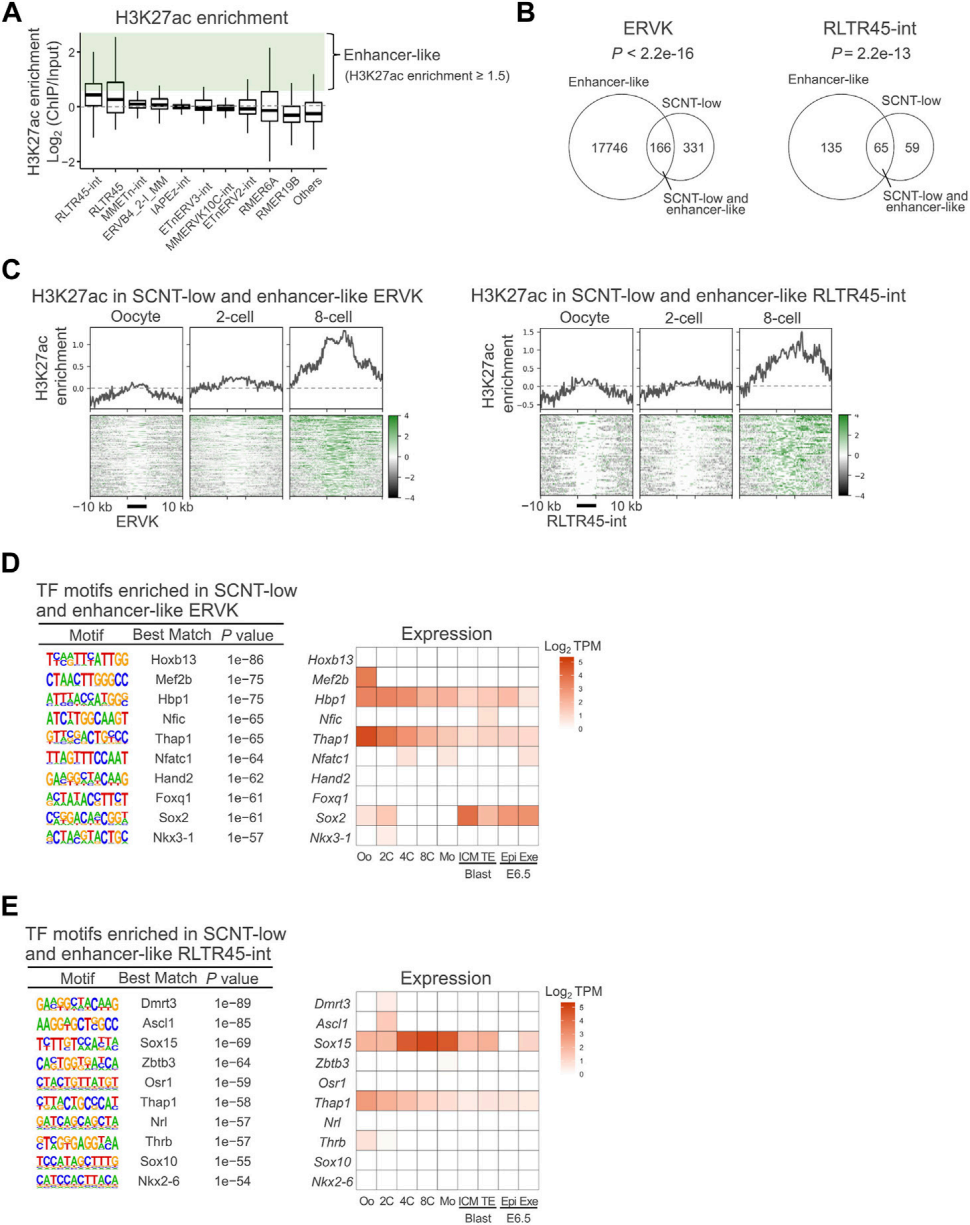
ERVKs suppressed in SCNT-derived embryos were rich in enhancer-like elements. **(A)** Box-and-whisker plots showing H3K27ac enrichment in the top 10 most highly expressed ERVK subfamilies. Enrichment is defined as the log_2_ ratio of ChIP data to the input. Enhancer-like ERVK loci were defined as those with H3K27ac enrichment (ChIP/Input) ≥ 1.5 (in green). Published H3K27ac ChIP-seq data (GSE72784) were analyzed. **(B)**Venn diagram showing the overlap between enhancer-like and SCNT-low ERVK (left) and RLTR45-int (right) loci. *p* values were calculated by Fisher’s exact test. **(C)** Heatmap and line graph showing H3K27ac enrichment at the SCNT-low, enhancer-like ERVK (left) and RLTR45-int (right) loci in MII oocytes and 2-cell and 8-cell embryos **(D)** Motif enrichment analysis by HOMER for SCNT-low, enhancer-like ERVK loci (left). Heatmap showing expression levels of transcription factors whose motif was enriched in SCNT-low, enhancer-like ERVK loci (right). **(E)** Motif enrichment analysis by HOMER for SCNT-low, enhancer-like RLTR45-int loci (left). Heatmap showing expression level of transcription factor whose motif was enriched in SCNT-low, enhancer-like RLTR45-int loci (right).

The positive correlations between the expression levels of ERVK elements, especially RLTR45-int, and their nearby genes might indicate that they have enhancer activity, as has been shown for other enhancer-like TEs (Fueyo et al., 2022). It is generally accepted that H3K27ac is increased at active enhancer sequences, contributing to elevated expressions of their regulating genes (Heintzman et al., 2009). Therefore, we examined whether the ERVK subfamilies were enriched with the enhancer marker H3K27ac by analyzing the published chromatin immunoprecipitation sequencing (ChIP-seq) data from metaphase stage II (MII) oocytes to 8-cell embryos (Dahl et al., 2016). As expected, RLTR45-int had the highest H3K27ac enrichment among the analyzed subfamilies (Figure 2A). We arbitrarily defined ‘enhancer-like’ loci as those with H3K27ac enrichment greater than 1.5 (ChIP/Input) for the subsequent analyses. Of the SCNT-low ERVK loci and SCNT-low RLTR45-int loci, 33% (166/497) and 52% (65/124), respectively, were defined as enhancer-like (Figure 2B). The SCNT-low, enhancer-like ERVK elements and RLTR45-int loci accumulated H3K27ac and H3K9ac, another enhancer marker, as the embryos developed (Figure 2C, Supplementary Figure S3C). On the other hand, the loci showed no enrichment of the promoter marker, H3K4me3, throughout preimplantation development (Supplementary Figure S3D). We examined whether the nearby genes around the SCNT-low, enhancer-like ERVK and RLTR45-int loci were also downregulated in SCNT embryos. As shown in Supplementary Figure S3E,F, these nearby genes at both 5–50 and 50–200 kb distances were downregulated (*r* = 0.14–0.32), although their correlations were not statistically different (*p* = 0.070 and 0.10, respectively), probably due to the smaller numbers of the nearby genes. By contrast, the downregulation of the nearby genes around the non-SCNT-low RLTR45-int loci was negligible or minor (*r* = 0.001–0.14) (Supplementary Figure S3G). These findings further confirmed that SCNT-low, enhancer-like ERVK/RLTR45-int loci were more resistant to reprogramming than other ERVK/RLTR45-int loci.

We next performed transcription factor motif analysis on SCNT-low, enhancer-like ERVK elements and RLTR45-int loci. In addition to the THAP1 motif that was generally enriched at ERVK loci, the motif for SOX15, a highly expressed gene at the morula stage, was specifically enriched at the RLTR45-int loci (Figures 2D,E). However, the *Sox15* gene was not repressed in SCNT embryos (Supplementary Figure S3H), indicating that the downregulation of the RLTR45-int loci in SCNT embryos may be more likely attributable to their repressive status *per se*, rather than decreased expressions of transcription factors. The *Sox15* gene is known to be highly conserved in eutherian mammals and is expressed in placental tissues, although aberration of this gene alone did not compromise embryo development in mice (Lee et al., 2004; Yamada et al., 2008). In summary, these data suggest that RLTR45-int exerts an enhancer-like function *via* transcription factors such as SOX15, thus inducing the transcription of genes important for postimplantation embryo development. It is possible that several transcription factors including SOX15 may act redundantly.

### Deletion of enhancer-like RLTR45-int results in decreased expression of nearby genes

**FIGURE 3:**
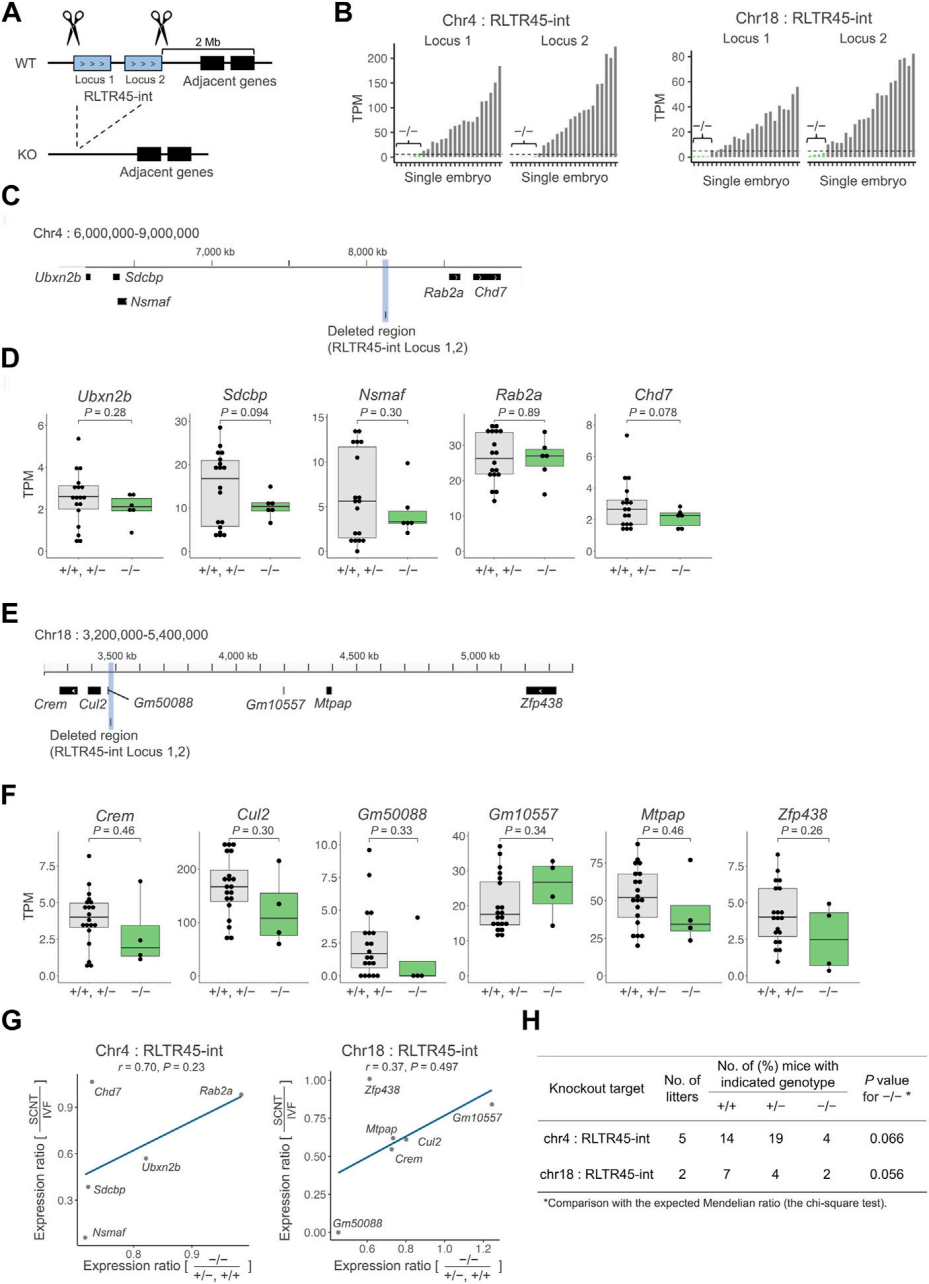
Deletion of SCNT-low and enhancer-like RLTR45-int loci caused decreased expressions of the adjacent genes. **(A)** Schematics of the experimental approach. Both ends of a target region including two SCNT-low, enhancer-like RLTR45-int loci on chromosome 4 or 18 were cut with the gRNAs-Cas9 complex to delete the entire region **(B)** Bar graphs showing expressions of two targeted RLTR45-int loci on chromosomes 4 (left) and 18 (right) after CRISPR/Cas9-mediated knockout. Homozygous knockout embryos were determined based on the no or negligible expression levels (TPM <5) of both RLTR45-int loci (depicted as “^−/−^”). **(C)** Genome browser view showing the deleted region and its adjacent genes which are expressed in morulae on chromosome 4. The deleted region is highlighted in gray. **(D)** Box-and-whisker plots comparing the expressions of the adjacent genes on chromosome 4 in putatively homozygous knockout (^−/−^) embryos and other embryos (^+/+^, ^+/−^). *p* values were calculated by a two-tailed *t*-test. **(E)** Genome browser view showing the deleted region and its adjacent genes which are expressed in morulae on chromosome 18. The deleted region is highlighted in gray. **(F)** Box-and-whisker plots comparing the expressions of the adjacent genes on chromosome 18 in putatively homozygous knockout embryos (^−/−^) and other embryos (^+/+^, ^+/−^). *p* values were calculated by a two-tailed *t*-test (right). **(G)** Scatterplot showing correlation of the decreased expression ratios of adjacent genes in SCNT (vs. IVF) and homozygous knockout (vs. ^+/+^ and ^+/−^) embryos on chromosomes 4 (left) and 18 (right). A tentative regression line without statistical values is shown because of the small number of samples. **(H)** *In vivo* development of embryos treated with CRISPR/Cas9 targeting RLTR45-int loci on chromosome 4 or 18. Embryos were produced by IVF using heterozygous females and males and transferred into pseudopregnant females. Live embryos were retrieved at E12.5 and genotyped for the targeting loci.

Next, we employed a gene knockout strategy to test whether RLTR45-int loci could regulate their nearby genes. We selected two sequential SCNT-low, enhancer-like RLTR45-int loci on chromosome 4 or 18, which were highly downregulated in SCNT embryos (adjusted *p* = 2.1e–31, 7.2e–29 on chromosome 4 and 2.7e–11, 7.6e–8 on chromosome 18) and associated with multiple SCNT-low nearby genes. The entire region of these two sequential RLTR45-int loci was deleted from the genome of IVF-derived embryos using the CRISPR/Cas9 system and the expression levels of their nearby genes were examined by single-embryo RNA-seq (Figure 3A). Of the embryos analyzed, six and four embryos targeted for RLTR45-int loci on chromosomes 4 and 18, respectively, were considered to be successfully knocked out based on the absence of or very low expression levels of the targeted RLTR45-int loci at the morula stage (Figure 3B, Supplementary Figure S4A). On chromosome 4, among the five expressed genes within 2 Mb of the RLTR45-int (see Figure 3C), 4 genes (*Ubxn2b*, *Sdcbp*, *Nsmaf*, and *Chd7*) were downregulated by deletion of the corresponding RLTR45-int loci, although not statistically significant (*p* = 0.078–0.30) (Figures 3C,D). On chromosome 18, five (*Crem*, *Cul2*, *Gm50088*, *Mtpap*, and *Zfp438*) of the six expressed genes within 2 Mb of the RLTR45-int (see Figure 3E) were downregulated by deletion of the corresponding RLTR45-int loci although not statistically significant (*p* = 0.26–0.46) (Figures 3E,F). Furthermore, positive correlations were observed between the decreased expression ratios of nearby genes by gene knockout and those by SCNT (vs. IVF) (Figure 3G, Supplementary Figure 4C,D). These results suggest that the RLTR45-int loci possess enhancer-like activities that regulate their nearby genes and that loss of such enhancer activity in SCNT-derived embryos caused downregulation of their nearby genes.

We also sought to determine whether the RLTR45-int deletion on chromosome 4 or 18 could affect postimplantation embryonic development. To this end, we generated mouse lines that lacked the RLTR45-int loci on chromosome 4 or 18. Embryos derived from IVF using heterozygous females and males were transferred into recipient females and examined at embryonic day (E) 12.5. The number of homozygous knockout embryos was smaller than expected, but with no statistical differences. Thus, there was no evidence that deletion of the RLTR45-int loci on chromosome 4 or 18 affects embryo development after implantation.

### RLTR45-int loci were enriched with reprogramming-resistant H3K9me3

**FIGURE 4:**
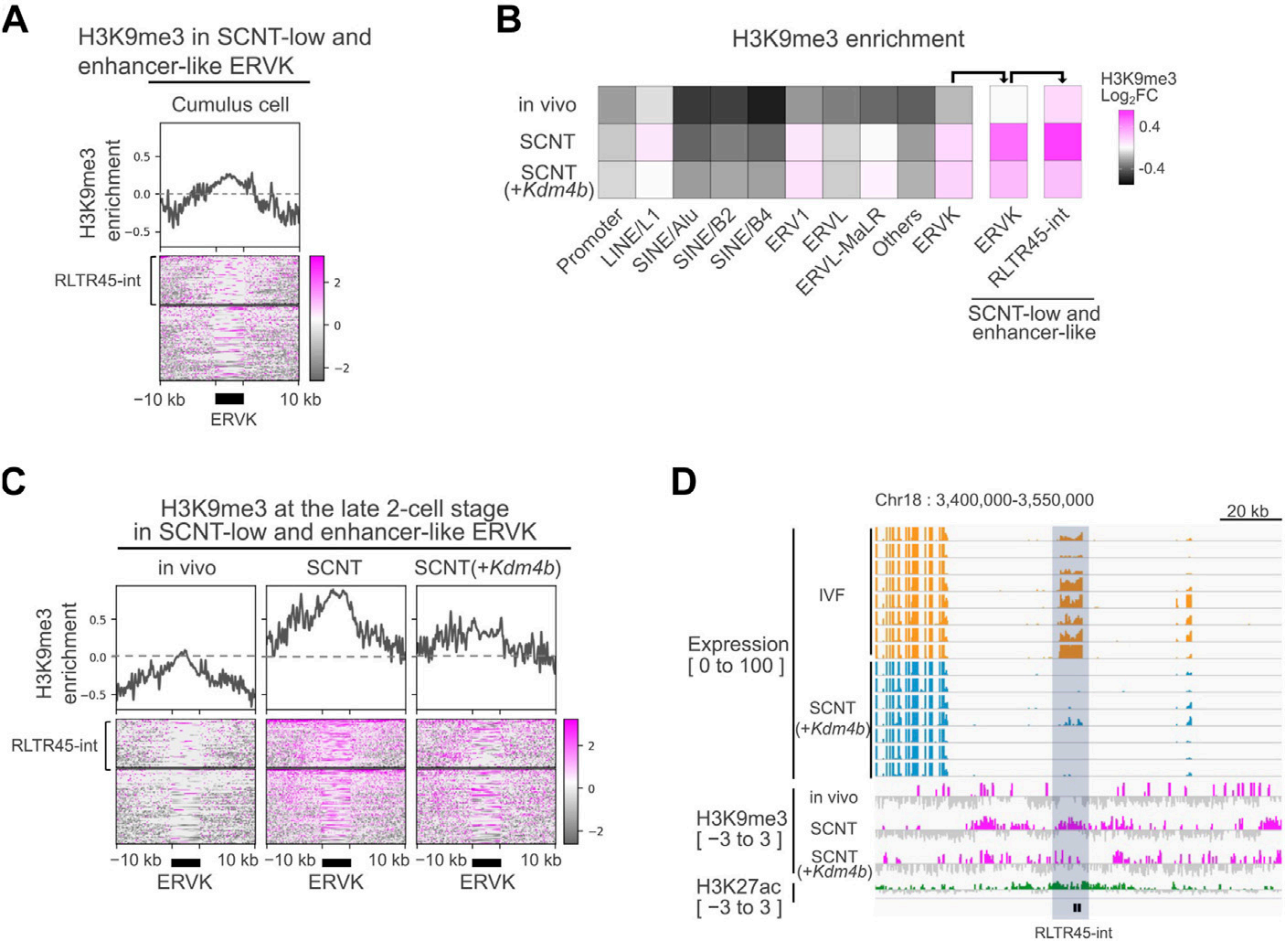
Donor cell-derived H3K9me3 was not fully reprogrammed at RLTR45-int loci. **(A)** Heatmap and line graph showing H3K9me3 enrichment at SCNT-low, enhancer-like ERVK loci in cumulus cells. The heatmap is divided into two parts: the upper represents the H3K9me3 enrichment of RLTR45-int loci and the lower represents that of the other ERVK loci. Published H3K9me3 ChIP-seq data (GSE70606) were analyzed. **(B)** Heatmap showing H3K9me3 enrichment of gene promoter, TE families, and SCNT-low, enhancer-like ERVK and RLTR45-int loci in 2-cell embryos. **(C)** Heatmap and line graph showing H3K9me3 enrichment at SCNT-low, enhancer-like ERVK loci *in vivo*, SCNT, and *Kdm4b*-mRNA-injected SCNT-derived 2-cell embryos. The heatmap is divided into two parts: the upper represents the H3K9me3 enrichment of RLTR45-int loci and the lower represents that of the other ERVK loci. **(D)** Genome browser view showing the expression levels in IVF (yellow) and SCNT (blue) morulae, H3K9me3 enrichment (red) *in vivo*, SCNT, *Kdm4b*-mRNA-injected SCNT 2-cell embryos, and H3K27ac enrichment (green) in 8-cell *in vivo* embryos around two SCNT-low, enhancer-like RLTR45-int loci on chromosome 18. The RLTR45-int region is highlighted in gray.

In somatic cells, the ERVK family is primarily repressed by H3K9me3 through the KRAB-ZFP/KAP1 system (Wolf and Macfarlan, 2015). Consistent with this, we found H3K9me3 enrichment at the SCNT-low, enhancer-like RLTR45-int loci in donor cumulus cells by analyzing H3K9me3 ChIP-seq data (Figure 4A) (Liu et al., 2016). Therefore, we investigated the possibility that defective reprogramming of the ERVK family including RLTR45-int following SCNT might be responsible for the somatic cell-derived H3K9me3 marks. We analyzed a publicly available database of H3K9me3 ChIP-seq data in vivo-derived embryos, SCNT-derived embryos, and *Kdm4b*-injected SCNT-derived embryos (Liu et al., 2016). Among the major TE families, ERV1 and ERVK had higher H3K9me3 levels in SCNT (GFP mRNA-injected)-derived embryos compared with *in vivo* embryos (Figure 4B). We found that SCNT-low, enhancer-like ERVKs had particularly high H3K9me3 levels in SCNT-derived embryos. Although these regions seemed to respond to *Kdm4b* treatment to some extent, they still had high H3K9me3 enrichment (Figures 4B,C). The expression levels of the RLTR45-int loci on chromosome 18 in IVF- and SCNT-derived embryos, together with their H3K9me3, and H3K27ac levels, were shown in a genome browser view (Figure 4D), which clearly displays the significant remnants of H3K9me3, unlike in vivo-derived embryos. These findings imply the existence of another epigenetic barrier repressing the RLTR45-int expression and enhancer activity of in SCNT embryos.

## Discussion

Here, we demonstrated that endogenous retroviral ERVK, especially its subfamily RLTR45-int, was specifically repressed in SCNT-derived morulae. We identified the RLTR45-int loci as putative enhancers because there was a significant correlation between the expression ratios (SCNT/IVF) of RLTR45-int loci and their nearby genes. Consistent with this observation, these loci were enriched with a typical enhancer marker H3K27ac, but not with the promoter marker H3K4me3. We further confirmed this assumption by CRISPR/Cas9-mediated knockout of RLTR45-int loci on two chromosomes, which resulted in downregulations of their nearby genes. The effects of the knockouts on the downregulations of these genes were not statistically significant (*p* > 0.05), but this might have been due to the relatively small proportions of successfully knocked-out embryos among the treated embryos. Indeed, there were significant correlations between the decreased expression ratios of adjacent genes in SCNT (*vs.* IVF) and homozygous knockout (*vs.* ^+/+^ and ^+/−^) embryos (Figure 3G). As far as we know, this is the first demonstration of enhancer-like TEs activated in late preimplantation embryos. It should be stressed that this finding could be achieved with our SCNT experimental system that can elicit dysregulation of specific genes in comparison with fertilized embryos. However, the enhancer-like TEs identified in this study might have been underestimated because we performed mRNA-seq studies that can detect only polyadenylated (polyA) RNAs. Given that most TE transcripts are thought to be devoid of polyA, employment of RNA-seq using random primers would enable us to identify new sets of TEs that might play specific roles during peri-implantation embryonic development.

Mammalian TEs with regulatory sequences originated from ancestral transposons that were integrated into the germline of the host genome. TEs, including ERVs, are suppressed by the epigenetic machinery such as repressive histone marks and DNA methylation to avoid their transposition and recombination capacity unless they are activated to play normal physiological roles (Kato et al., 2018; He et al., 2019). In late preimplantation mouse embryos, different layers of repressive histone marks cooperatively silence harmful TEs because their genomic DNA is largely hypomethylated (Hatanaka et al., 2015; Wang et al., 2018). Here, we found that ERVKs including the RLTR45-int loci escape this histone-mediated silencing and thus at least some of them can exert their enhancer-like functions at the morula stage. Paradoxically, ERVKs are relatively enriched with repressive H3K9me3 marks compared with other TEs (Figure 4B). This is in accordance with a finding that the genome of preimplantation embryos contains large H3K9me3 domains occupying LTR regions (such as ERVL and ERVK), but not LINE or SINE regions (Wang et al., 2018). Therefore, we do not know how ERVKs escape the wave of genome-wide TE silencing in a natural setting. According to our study (Hatanaka et al., 2015), TEs in preimplantation embryos are predominantly silenced by H4K20me3 rather than by H3K9me3, based on the results from knockdown of different histone methyltransferases. During preimplantation development, initial H3K9me3 deposited by Suv39h2 is not repressive, and later Suv39h1-mediated H3K9me3 deposition, which is accompanied by H4K20me3, forms constitutively repressive heterochromatin (Burton et al., 2020). It is known that most morulae undergo arrested development by de-repression of many TEs induced by knockdown of CAF-1, a histone chaperone conveying H3.1 as well as H4. It is possible that H4K20me3 is lacking in the ERVK regions of preimplantation embryos. This question should be clarified in future studies.

Another important finding in this study is that RLTR45-int loci were further enriched with H3K9me3 in SCNT-derived embryos. This aberrant H3K9me3 deposition in RLTR45-int is most likely a carry-over from the nucleus-donor cumulus cells (Figure 4A). However, unlike MERVL in SCNT 2-cell embryos (Matoba et al., 2014), treatment with the H3K9 demethylase Kdm4d failed to activate RLTR45-int in SCNT-derived embryos. This might have been because of the abundance of H3K9me3 or the coexistence of other reprogramming-resistant epigenetic barriers, which may lead to formation of strongly heterochromatinized regions at the RLTR45-int loci. We estimate that there are about 400 RLTR45-int loci in the mouse genome, although the precise annotation of the RNA-seq products to specific loci is technically difficult due to their sequence similarities. The multicopy RLTR45-int loci act as enhancers to regulate a number of downstream genes at the morula stage, suggesting that this large-scale gene activating cascade might play critical roles in subsequent developmental events. Abnormal RLTR45-int silencing in SCNT morulae helps explain the frequent death of SCNT-derived embryos shortly after implantation. As RLTR45-int was preferentially expressed in the inner cell mass rather than trophectoderm in blastocysts (Figure 1F), its downregulation might affect the development of embryonic tissue (epiblast and/or primitive endoderm) in SCNT-derived embryos. In this study, we successfully deleted two sequential RLTR45-int loci on chromosome 4 and 18 that regulate four to five downstream genes, respectively, in IVF-derived embryos. As far as we observed, these individual knockouts seemed to have no adverse effects on subsequent embryonic development (Figure 3H). It is probable that a set of genes regulated by multiple RLTR45-int loci may have functional redundancy that makes knockout of a single locus negligible. To overcome this, it is essential to delete all or many of RLTR45-int by CRISPR/Cas9 one at a time, but it is difficult to design appropriate guide (g)RNAs that specifically target the RLTR45-int sequences. Low-specific gRNAs would cause frequent off-target mutations that would irreversibly damage the genome. Another approach would be the “rescue” of SCNT-derived embryos by activation of RLTR45-int. If some epigenetic treatments allow global activation of RLTR45-int and improve the development of SCNT-derived embryos, then conversely the results indicate that RLTR45-int is important for postimplantation embryonic development. Further comprehensive epigenetic analyses of SCNT-derived embryos at peri-implantation stages and the development of more refined epigenetic modulation technology should reveal this point in future studies.

## Materials and methods

### Animals

All animal experiments were approved by the Animal Experimentation Committees at the RIKEN Tsukuba Institute and were performed in accordance with the committees’ guidelines. Animals were housed under controlled temperature (24 ± 1°C), humidity (55% ± 2%), and a lighting condition (daily light period, 07:00–21:00). They were provided with food and water *ad libitum* and maintained under specific pathogen-free conditions.

### *In vitro* fertilization

C57BL/6N (Japan SLC, Shizuoka, Japan) female mice at 4–20 weeks of age were superovulated by injection of 7.5 IU equine chorionic gonadotropin (eCG; PMSG, Sankyo Co. Ltd., Tokyo, Japan) or anti-inhibin serum (100 μL per mouse; Central Research Co. Ltd., Tokyo, Japan) (Hasegawa et al., 2022), followed 46–52 h later by injection of 7.5 IU human chorionic gonadotropin (hCG; Puberogen, Sankyo). Female mice were euthanized by cervical dislocation 16 h after the hCG injection, and cumulus–oocyte complexes were collected from the ampullae of excised oviducts and placed in 100-μL droplets of human tubal fluid (HTF) medium. Spermatozoa were collected from the cauda epididymiis of adult DBA2 male mice (Japan SLC) and preincubated for at least 30 min in a 400-μL droplet of HTF. After preincubation, 3–5 μL of the sperm suspension was added to the oocyte cultures. At 3 h post-insemination (hpi), morphologically normal fertilized embryos were collected and cultured in potassium simplex optimized medium (KSOM). All incubations were performed at 37°C under 5% CO_2_ in air.

### *In vitro* transcription of mRNA

The procedure of mRNA synthesis was carried out as described (Matoba et al., 2014). After a pcDNA3.1 plasmid carrying the full-length mouse *Kdm4d* sequence (61553; Addgene, Watertown, MA, United States) was linearized by XbaI, it was used as a template for *in vitro* transcription using mMESSAGE mMACHINE T7 ULTRA Transcription kits (AM1345; Thermo Fisher Scientific, Waltham, MA, United States). The synthesized mRNA was diluted to 1500 ng/μL in nuclease-free water and stored at −80°C until use.

### Somatic cell nuclear transfer and *Kdm4d* mRNA injection

SCNT was done as described (Matoba et al., 2018). Cumulus cells used as nuclear donors were collected from wild-type and *Xist* heterozygous knockout female mice with a BDF1 (C57BL/6N × DBA2) genetic background. Recipient oocytes were collected from BDF1 female mice following superovulation as above, and placed in KSOM droplets containing 0.1% bovine testicular hyaluronidase (385931; Calbiochem, La Jolla, CA, United States) to remove cumulus cells. The cumulus-free oocytes were enucleated in Hepes-buffered KSOM containing 7.5 μg/ml cytochalasin B using a Piezo-driven pipette (PMM-150FU; Prime Tech, Ibaraki, Japan). After being cultured in fresh KSOM for 1 h, the enucleated oocytes were injected with cumulus cell nuclei in Hepes-buffered KSOM at room temperature. After 1 h culture, the injected oocytes were activated by culture in a Ca^2+^-free KSOM containing 3 mM strontium chloride and 5 μg/ml cytochalasin B for 1 h. The reconstructed oocytes were cultured in KSOM containing 5 μg/ml cytochalasin B for 5 h. After being washed thoroughly, SCNT-derived embryos were injected with 10 pL of 1500 ng/μL mouse *Kdm4d* mRNA at 5–6 h postactivation (hpa).

### Design and synthesis of guide RNAs

The gRNAs were synthesized using GeneArt Precision gRNA Synthesis kits (A29377; Thermo Fisher Scientific). Briefly, two gRNAs were designed by a software tool (https://www.benchling.com) to target the 5′ and 3′ regions outside the specific RLTR45-int loci on each of mouse chromosomes 4 and 18. DNA templates of gRNAs were assembled by polymerase chain reaction (PCR) using specific primer sets, and then gRNAs were transcribed *in vitro* based on the templates. The gRNAs used in this study are listed in Supplementary Table S1.

### CRISPR/Cas9-mediated deletion of RLTR45-int

IVF was performed as described above, with slight modifications. Spermatozoa were collected from C57BL/6N male mice and 3–5 μL of the sperm suspension was added to oocyte cultures containing 1 mM glutathione (G6013-5G, Sigma-Aldrich, St. Louis, MO, United States) to improve the fertilization rate. At 6–7 hpi, 20–30 embryos were placed into a chamber with 5 μL of Opti-MEM (51985062; Thermo Fisher Scientific) containing 200 ng/μL Cas9 (1081058; Integrated DNA Technologies Inc, Coralville, IA, United States) and 100 ng/μL gRNA. They were electroporated with a 1-mm gap electrode (CUY501P1; Nepa Gene, Chiba, Japan) in a NEPA21 Super Electroporator (Nepa Gene). The pulses used for electroporation were voltage 25–30 V, pulse width 2 ms, pulse interval 50 ms, and number of pulses 4. The first and second transfer pulses were voltage 10 V, pulse width 2 ms, pulse interval 50 ms, and number of pulses 4. Embryos cultured *in vitro* were collected at 69 hpi for RNA-seq experiments, and the remaining embryos were transferred into pseudopregnant ICR female mice. F0 founder mice carrying mutations were used for establishment of knockout mouse lines. IVF embryos derived from F1 generation heterozygous females and males were transferred into pseudopregnant mice that were mated with vasectomized male mice overnight before embryo transfer. On E12.5, embryos were retrieved from the uteri to assess the *in vivo* developmental ability of embryos based on the genotype.

### Genotyping

Genomic DNA was extracted from tail tip or fetal tissues using a Wizard Genomic DNA Purification Kit (A1120; Promega, Madison, WI, United States). Primers were designed to amplify the deleted regions. PCR was performed using TaKaRa Ex Taq Hot Start Version (RR006A; Takara Bio Inc, Shiga, Japan) under the following conditions: 1 cycle of 98°C for 10 s; 30 cycles of 98°C for 10 s, 60°C for 30 s, and 72°C for 40 s; and 1 final step of 72°C for 1 min. Sanger sequencing of the PCR products was outsourced to Eurofins Genomics Inc. (Tokyo, Japan). Primers used in this study are listed in Supplementary Table S1.

### RNA-seq library preparation

IVF- and SCNT-derived embryos were collected at 69–70 hpi/hpa, washed twice in 0.05% bovine serum albumin in phosphate-buffered saline, and flash-frozen in liquid nitrogen. After thawing, polyadenylated RNAs were reverse-transcribed and amplified, using SMART-Seq v4 kits (R00752; Takara Bio Inc.). Embryos electroporated with Cas9 and gRNAs were collected at 69–70 hpi, and the sequence libraries were synthesized using SMART-Seq HT kits (R400748; Takara Bio Inc.). The quality of sequence libraries was examined using a 2100 Bioanalyzer with High Sensitivity DNA kits (5067–4626; Agilent Technologies, Santa Clara, CA, United States). Paired-end 150-bp sequencing was performed on a HiSeq X platform (Illumina, San Diego, CA, United States).

### RNA-seq analysis

Adapter sequences and low-quality reads were removed with Trimmomatic (version 0.36) (Bolger et al., 2014). The resulting sequence reads were aligned uniquely to the mm10 mouse genome using STAR aligner software (version 2.7.5c) (Dobin et al., 2013) with parameters as follows: “—alignIntronMin 20—alignIntronMax 1000000—alignMatesGapMax 1000000— alignSJoverhangMin 8—alignSJDBoverhangMin 1—twopass Mode Basic—readFilesCommand zcat—outFilterType BySJout —outFilterMultimapNmax 1—outFilterMismatchNmax 999— outFilterMismatchNoverReadLmax 0—outSAMtype BAM Unsorted—winAnchorMultimapNmax 50”. The mapped reads were counted using featureCounts (Liao et al., 2014). The expression levels of genes were calculated with normalized transcripts per kilobase million (TPM) data, and the expression level of TE locus was calculated with normalized TPM and counts per million (CPM). Differentially expressed genes and TE loci were analyzed by the DESeq2 package (version 1.16.1) (Love et al., 2014). Expressed transcripts with at least one read on average were used for analysis. Principal component analysis (PCA) was performed using RLE-normalized counts by DEseq2. The Spearman correlation coefficient of expression level was computed to indicate the correlation between duplicates. Gene ontology enrichment analysis was done in Metascape (version 3.5). Sex chromosomes were excluded from this analysis; this is because IVF-derived embryos include male and female cells while all SCNT-derived embryos are female, so the ratio of X and Y chromosomes is different between the two sets of embryos.

### Chromatin immunoprecipitation sequencing analysis

Adapter sequences and low-quality reads were removed with Trimmomatic (version 0.36) (Bolger et al., 2014). The resulting sequence reads were aligned to the mm10 mouse genome using Bowtie2 aligner software (version 2.4.4) (Langmead and Salzberg, 2012), not allowing a single mismatch in the alignment. Reads from PCR duplicates were filtered using Samtools (version 1.10) (Li et al., 2009) “markdup” with the option “−r”, and then the filtered reads with the “XS” tag were also filtered to extract only uniquely mapped reads. Read counts of replicates were down-sampled until a similar read count was achieved using “divide_bam.py” in RSeQC (Wang et al., 2012) before replicates were merged using Samtools “merge”, so that replicates contributed equally to the merged file. The mouse genome (mm10) was divided into 2-kb bins in a sliding window of 1 kb. Promoters were defined as −2,000– + 500 bp relative to the transcription start site. Read counts for 2-kb bin windows, TE loci for ChIP and input were obtained using “featureCounts” (Liao et al., 2014). Read counts for promoters were obtained from 2-kb windows with maximum overlaps with defined promotes. ChIP and input reads per kilobase per million reads (RPKM) values of TE loci were calculated using the following formula: read count for TE loci/(length of TE loci/1,000 × total read count for 2-kb bin windows/1,000,000). Relative ChIP enrichment of TE loci was calculated by dividing the ChIP (RPKM + 0.5) value of TE loci by the input (RPKM + 0.5) value of TE loci, followed by log_2_ transformation. Relative ChIP enrichment of promoters was obtained in the same way as above. Heatmaps were drawn using “computeMatrix” and “plotHeatmap” in deepTools (version 3.4.3). Enrichment of known motifs within enhancer-like ERVK loci was identified using HOMER (version 4.10) (Heinz et al., 2010) using the function “findMotifsGenome.pl -size given” option. Sex chromosomes were excluded from this analysis (see the “RNA-seq analysis” section above).

### Statistical analysis

Statistical analyses were performed using R (version 4.0.4; https://www.r-project.org). *p* values were calculated using two-tailed *t*-tests (Figures 1C,E, 3D,F). Spearman correlation coefficients were calculated using the “cor” function in R (Figures 1J, 3G, Supplementary Figure S3F,G). In the *in vivo* development experiments, the proportions of mice with three genotypes were each compared to the expected Mendelian ratios by the chi-square test (Figure 3H). *p* values were calculated by applying Fisher’s exact test (Figure 2B). *p* values <0.05 were considered to be statistically significant.

## Data Availability

TThe original contributions presented in the study are publicly available. This data can be found here: https://www.ncbi.nlm.nih.gov/geo/query/acc.cgi?acc=GSE214878.

## References

[B1] Akter M. S. Hada M. Shikata D. Watanabe G. Ogura A. Matoba S. (2021). CRISPR/Cas9-based genetic screen of SCNT-reprogramming resistant genes identifies critical genes for male germ cell development in mice. Sci. Rep. 11, 15438. 10.1038/s41598-021-94851-9 34326397 PMC8322354

[B2] Bolger A. M. Lohse M. Usadel B. (2014). Trimmomatic: A flexible trimmer for illumina sequence data. Bioinformatics 30, 2114–2120. 10.1093/bioinformatics/btu170 24695404 PMC4103590

[B3] Burton A. Brochard V. Galan C. Ruiz-Morales E. R. Rovira Q. Rodriguez-Terrones D. (2020). Heterochromatin establishment during early mammalian development is regulated by pericentromeric RNA and characterized by non-repressive H3K9me3. Nat. Cell Biol. 22, 767–778. 10.1038/s41556-020-0536-6 32601371 PMC7610380

[B4] Chuong E. B. Rumi M. A. K. Soares M. J. Baker J. C. (2013). Endogenous retroviruses function as species-specific enhancer elements in the placenta. Nat. Genet. 45, 325–329. 10.1038/ng.2553 23396136 PMC3789077

[B5] Dahl J. A. Jung I. Aanes H. Greggains G. D. Manaf A. Lerdrup M. (2016). Broad histone H3K4me3 domains in mouse oocytes modulate maternal-to-zygotic transition. Nature 537, 548–552. 10.1038/nature19360 27626377 PMC6283663

[B6] Dobin A. Davis C. A. Schlesinger F. Drenkow J. Zaleski C. Jha S. (2013). Star: Ultrafast universal RNA-seq aligner. Bioinformatics 29, 15–21. 10.1093/bioinformatics/bts635 23104886 PMC3530905

[B7] Fueyo R. Judd J. Feschotte C. Wysocka J. (2022). Roles of transposable elements in the regulation of mammalian transcription. Nat. Rev. Mol. Cell Biol. 23, 481–497. 10.1038/s41580-022-00457-y 35228718 PMC10470143

[B8] Gao R. Wang C. Gao Y. Xiu W. Chen J. Kou X. (2018). Inhibition of aberrant DNA re-methylation improves post-implantation development of somatic cell nuclear transfer embryos. Cell Stem Cell 23, 426–435. e5. 10.1016/j.stem.2018.07.017 30146410

[B9] Ge S. X. (2017). Exploratory bioinformatics investigation reveals importance of “junk” DNA in early embryo development. BMC Genomics 18, 200. 10.1186/s12864-017-3566-0 28231763 PMC5324221

[B10] Hada M. Miura H. Tanigawa A. Matoba S. Inoue K. Ogonuki N. (2022). Highly rigid H3.1/H3.2-H3K9me3 domains set a barrier for cell fate reprogramming in trophoblast stem cells. Genes Dev. 36, 84–102. 10.1101/gad.348782.121 34992147 PMC8763053

[B11] Hasegawa A. Mochida K. Nakamura A. Miyagasako R. Ohtsuka M. Hatakeyama M. (2022). Use of anti-inhibin monoclonal antibody for increasing the litter size of mouse strains and its application to *in vivo*-genome editing technology. Biol. Reprod. 107, 605–618. 10.1093/biolre/ioac068 35368067 PMC9382380

[B12] Hatanaka Y. Inoue K. Oikawa M. Kamimura S. Ogonuki N. Kodama E. N. (2015). Histone chaperone CAF-1 mediates repressive histone modifications to protect preimplantation mouse embryos from endogenous retrotransposons. Proc. Natl. Acad. Sci. U. S. A. 112, 14641–14646. 10.1073/pnas.1512775112 26546670 PMC4664303

[B13] He J. Fu X. Zhang M. He F. Li W. Abdul M. M. (2019). Transposable elements are regulated by context-specific patterns of chromatin marks in mouse embryonic stem cells. Nat. Commun. 10, 34. 10.1038/s41467-018-08006-y 30604769 PMC6318327

[B14] Heintzman N. D. Hon G. C. Hawkins R. D. Kheradpour P. Stark A. Harp L. F. (2009). Histone modifications at human enhancers reflect global cell-type-specific gene expression. Nature 459, 108–112. 10.1038/nature07829 19295514 PMC2910248

[B15] Heinz S. Benner C. Spann N. Bertolino E. Lin Y. C. Laslo P. (2010). Simple combinations of lineage-determining transcription factors prime *cis*-regulatory elements required for macrophage and B cell identities. Mol. Cell 38, 576–589. 10.1016/j.molcel.2010.05.004 20513432 PMC2898526

[B16] Inoue A. Jiang L. Lu F. Zhang Y. (2017). Genomic imprinting of Xist by maternal H3K27me3. Genes Dev. 31, 1927–1932. –1932. 10.1101/gad.304113.117 29089420 PMC5710138

[B17] Inoue K. Kohda T. Sugimoto M. Sado T. Ogonuki N. Matoba S. (2010). Impeding Xist expression from the active X chromosome improves mouse somatic cell nuclear transfer. Science 330, 496–499. 10.1126/SCIENCE.1194174/SUPPL_FILE/INOUE-SOM 20847234

[B18] Inoue K. Ogonuki N. Kamimura S. Inoue H. Matoba S. Hirose M. (2020). Loss of H3K27me3 imprinting in the Sfmbt2 miRNA cluster causes enlargement of cloned mouse placentas. Nat. Commun. 11, 2150. 10.1038/s41467-020-16044-8 32358519 PMC7195362

[B19] Inoue K. Oikawa M. Kamimura S. Ogonuki N. Nakamura T. Nakano T. (2015). Trichostatin A specifically improves the aberrant expression of transcription factor genes in embryos produced by somatic cell nuclear transfer. Sci. Rep. 5, 10127. 10.1038/srep10127 25974394 PMC4431350

[B20] Kamimura S. Inoue K. Mizutani E. Kim J.-M. Inoue H. Ogonuki N. (2021). Improved development of mouse somatic cell nuclear transfer embryos by chlamydocin analogues, class I and IIa histone deacetylase inhibitors. Biol. Reprod. 105, 543–553. 10.1093/biolre/ioab096 33982061 PMC8335354

[B21] Kato M. Takemoto K. Shinkai Y. (2018). A somatic role for the histone methyltransferase Setdb1 in endogenous retrovirus silencing. Nat. Commun. 9, 1683. 10.1038/s41467-018-04132-9 29703894 PMC5923290

[B22] Kigami D. Minami N. Takayama H. Imai H. (2003). MuERV-L is one of the earliest transcribed genes in mouse one-cell embryos. Biol. Reprod. 68, 651–654. 10.1095/biolreprod.102.007906 12533431

[B23] Kishigami S. Wakayama S. Van Thuan N. Ohta H. Mizutani E. Hikichi T. (2006). Production of cloned mice by somatic cell nuclear transfer. Nat. Protoc. 1, 125–138. 10.1038/nprot.2006.21 17406224

[B24] Langmead B. Salzberg S. L. (2012). Fast gapped-read alignment with Bowtie 2. Nat. Methods 9, 357–359. 10.1038/nmeth.1923 22388286 PMC3322381

[B25] Lee H.-J. Göring W. Ochs M. Mühlfeld C. Steding G. Paprotta I. (2004). Sox15 is required for skeletal muscle regeneration. Mol. Cell. Biol. 24, 8428–8436. 10.1128/MCB.24.19.8428-8436.2004 15367664 PMC516755

[B26] Li H. Handsaker B. Wysoker A. Fennell T. Ruan J. Homer N. (2009). The sequence alignment/map format and SAMtools. Bioinformatics 25, 2078–2079. 10.1093/bioinformatics/btp352 19505943 PMC2723002

[B27] Liao Y. Smyth G. K. Shi W. (2014). featureCounts: an efficient general purpose program for assigning sequence reads to genomic features. Bioinformatics 30, 923–930. 10.1093/bioinformatics/btt656 24227677

[B28] Liu W. Liu X. Wang C. Gao Y. Gao R. Kou X. (2016). Identification of key factors conquering developmental arrest of somatic cell cloned embryos by combining embryo biopsy and single-cell sequencing. Cell Discov. 2, 16010. 10.1038/celldisc.2016.10 27462457 PMC4897595

[B29] Love M. I. Huber W. Anders S. (2014). Moderated estimation of fold change and dispersion for RNA-seq data with DESeq2. Genome Biol. 15, 550. 10.1186/s13059-014-0550-8 25516281 PMC4302049

[B30] Macfarlan T. S. Gifford W. D. Driscoll S. Lettieri K. Rowe H. M. Bonanomi D. (2012). Embryonic stem cell potency fluctuates with endogenous retrovirus activity. Nature 487, 57–63. 10.1038/nature11244 22722858 PMC3395470

[B31] Matoba S. Inoue K. Kohda T. Sugimoto M. Mizutani E. Ogonuki N. (2011). RNAi-mediated knockdown of Xist can rescue the impaired postimplantation development of cloned mouse embryos. Proc. Natl. Acad. Sci. U. S. A. 108, 20621–20626. 10.1073/pnas.1112664108 22065773 PMC3251083

[B32] Matoba S. Kozuka C. Miura K. Inoue K. Kumon M. Hayashi R. (2022). Noncanonical imprinting sustains embryonic development and restrains placental overgrowth. Genes Dev. 36, 483–494. 10.1101/gad.349390.122 35483741 PMC9067403

[B33] Matoba S. Liu Y. Lu F. Iwabuchi K. A. Shen L. Inoue A. (2014). Embryonic development following somatic cell nuclear transfer impeded by persisting histone methylation. Cell 159, 884–895. 10.1016/j.cell.2014.09.055 25417163 PMC4243038

[B34] Matoba S. Wang H. Jiang L. Lu F. Iwabuchi K. A. Wu X. (2018). Loss of H3K27me3 imprinting in somatic cell nuclear transfer embryos disrupts post-implantation development. Cell Stem Cell 23, 343–354. e5. 10.1016/j.stem.2018.06.008 30033120 PMC6326833

[B35] Matoba S. Zhang Y. (2018). Somatic cell nuclear transfer reprogramming: Mechanisms and applications. Cell Stem Cell 23, 471–485. 10.1016/j.stem.2018.06.018 30033121 PMC6173619

[B36] Modzelewski A. J. Shao W. Chen J. Lee A. Qi X. Noon M. (2021). A mouse-specific retrotransposon drives a conserved Cdk2ap1 isoform essential for development. Cell 184, 5541–5558.e22. e22. 10.1016/j.cell.2021.09.021 34644528 PMC8787082

[B37] Ogura A. Matoba S. Inoue K. (2021). 25th Anniversary of Cloning by Somatic-Cell Nuclear Transfer: Epigenetic abnormalities associated with somatic cell nuclear transfer. Reproduction 162, F45–F58. 10.1530/rep-21-0013 33635828

[B38] Oikawa M. Inoue K. Shiura H. Matoba S. Kamimura S. Hirose M. (2014). Understanding the X chromosome inactivation cycle in mice: A comprehensive view provided by nuclear transfer. Epigenetics 9, 204–211. 10.4161/epi.26939 24172050 PMC3962530

[B39] Peaston A. E. Evsikov A. V. Graber J. H. de Vries W. N. Holbrook A. E. Solter D. (2004). Retrotransposons regulate host genes in mouse oocytes and preimplantation embryos. Dev. Cell 7, 597–606. 10.1016/j.devcel.2004.09.004 15469847

[B40] Rybouchkin A. Kato Y. Tsunoda Y. (2006). Role of histone acetylation in reprogramming of somatic nuclei following nuclear transfer. Biol. Reprod. 74, 1083–1089. 10.1095/biolreprod.105.047456 16481594

[B41] Simmet K. Wolf E. Zakhartchenko V. (2020). Manipulating the epigenome in nuclear transfer cloning: Where, when and how. Int. J. Mol. Sci. 22, E236. 10.3390/ijms22010236 PMC7794987 33379395

[B42] Todd C. D. Deniz Ö. Taylor D. Branco M. R. (2019). Functional evaluation of transposable elements as enhancers in mouse embryonic and trophoblast stem cells. Elife 8, e44344. 10.7554/eLife.44344 31012843 PMC6544436

[B43] Trojer P. Reinberg D. (2007). Facultative heterochromatin: Is there a distinctive molecular signature? Mol. Cell 28, 1–13. 10.1016/j.molcel.2007.09.011 17936700

[B44] Wakayama T. Perry A. C. Zuccotti M. Johnson K. R. Yanagimachi R. (1998). Full-term development of mice from enucleated oocytes injected with cumulus cell nuclei. Nature 394, 369–374. 10.1038/28615 9690471

[B45] Wang C. Liu X. Gao Y. Yang L. Li C. Liu W. (2018). Reprogramming of H3K9me3-dependent heterochromatin during mammalian embryo development. Nat. Cell Biol. 20, 620–631. 10.1038/s41556-018-0093-4 29686265

[B46] Wang L.-Y. Li Z.-K. Wang L.-B. Liu C. Sun X.-H. Feng G.-H. (2020). Overcoming intrinsic H3K27me3 imprinting barriers improves post-implantation development after somatic cell nuclear transfer. Cell Stem Cell 27, 315–325. e5. 10.1016/j.stem.2020.05.014 32559418

[B47] Wang L. Wang S. Li W. (2012). RSeQC: Quality control of RNA-seq experiments. Bioinformatics 28, 2184–2185. 10.1093/bioinformatics/bts356 22743226

[B48] Wolf G. Macfarlan T. S. (2015). Revealing the complexity of retroviral repression. Cell 163, 30–32. 10.1016/j.cell.2015.09.014 26406368 PMC6309249

[B49] Xie Z. Zhang W. Zhang Y. (2022). Loss of Slc38a4 imprinting is a major cause of mouse placenta hyperplasia in somatic cell nuclear transferred embryos at late gestation. Cell Rep. 38, 110407. 10.1016/j.celrep.2022.110407 35196486 PMC8919768

[B50] Yamada K. Kanda H. Aihara T. Takamatsu N. Shiba T. Ito M. (2008). Mammalian Sox15 gene: Promoter analysis and implications for placental evolution. Zool. Sci. 25, 313–320. 10.2108/zsj.25.313 18393569

[B51] Yang G. Zhang L. Liu W. Qiao Z. Shen S. Zhu Q. (2021). Dux-mediated corrections of aberrant H3K9ac during 2-cell genome activation optimize efficiency of somatic cell nuclear transfer. Cell Stem Cell 28, 150–163.e5. e5. 10.1016/j.stem.2020.09.006 33049217

[B52] Yang L. Song L. Liu X. Bai L. Li G. (2018). KDM 6A and KDM 6B play contrasting roles in nuclear transfer embryos revealed by MERVL reporter system. EMBO Rep. 19, e46240. 10.15252/embr.201846240 30389724 PMC6280793

[B53] Zhang B. Zheng H. Huang B. Li W. Xiang Y. Peng X. (2016). Allelic reprogramming of the histone modification H3K4me3 in early mammalian development. Nature 537, 553–557. 10.1038/nature19361 27626382

[B54] Zhang K. Wu D.-Y. Zheng H. Wang Y. Sun Q.-R. Liu X. (2020). Analysis of genome architecture during SCNT reveals a role of cohesin in impeding minor ZGA. Mol. Cell 79, 234–250. e9. 10.1016/j.molcel.2020.06.001 32579944

[B55] Zhou C. Wang Y. Zhang J. Su J. An Q. Liu X. (2019). H3K27me3 is an epigenetic barrier while KDM6A overexpression improves nuclear reprogramming efficiency. FASEB J. 33, 4638–4652. 10.1096/fj.201801887R 30673507

