# Supplementary material for: Suppression of endogenous retroviral enhancers in mouse embryos derived from somatic cell nuclear transfer
Source: Front Genet. 2022 Nov 8;13:1032760. doi: 10.3389/fgene.2022.1032760 (PMC9681155; doi:10.3389/fgene.2022.1032760)
Supplement: Supplementary file 1 [file DataSheet1.pdf]

Suppression of endogenous retroviral enhancers in mouse embryos derived from somatic cell nuclear transfer

Supplementary Material

Daiki Shikata[1,2], Shogo Matoba[1,3], Masashi Hada[1,4], Akihiko Sakashita[5], Kimiko Inoue[1,2], and Atsuo Ogura[1,2,6*]

[1]*Bioresource Engineering Division, BioResource Research Center, RIKEN, Tsukuba, Ibaraki 305-0074, Japan*

[2]*Graduate school of Life and Environmental Sciences, University of Tsukuba, Tsukuba, Ibaraki 305-8577, Japan.*

[3]*Cooperative Division of Veterinary Sciences, Tokyo University of Agriculture and Technology, Fuchu, Tokyo 183-8509, Japan*

[4]*Laboratory of Pathology and Development, Institute for Quantitative Biosciences, The University of Tokyo, Tokyo, 113-0032, Japan.*

[5]*Department of Molecular Biology, Keio University School of Medicine, Tokyo 160-8582, Japan*

[6]*RIKEN Cluster for Pioneering Research, Hirosawa, Wako, Saitama 351-0198, Japan*

[*]Correspondence: Atsuo Ogura, D.V.M., PhD

RIKEN BioResource Research Center, Ibaraki 305-0074, Japan

Tel.: 81-29-836-9165; e-mail: ogura@rtc.riken.go.jp

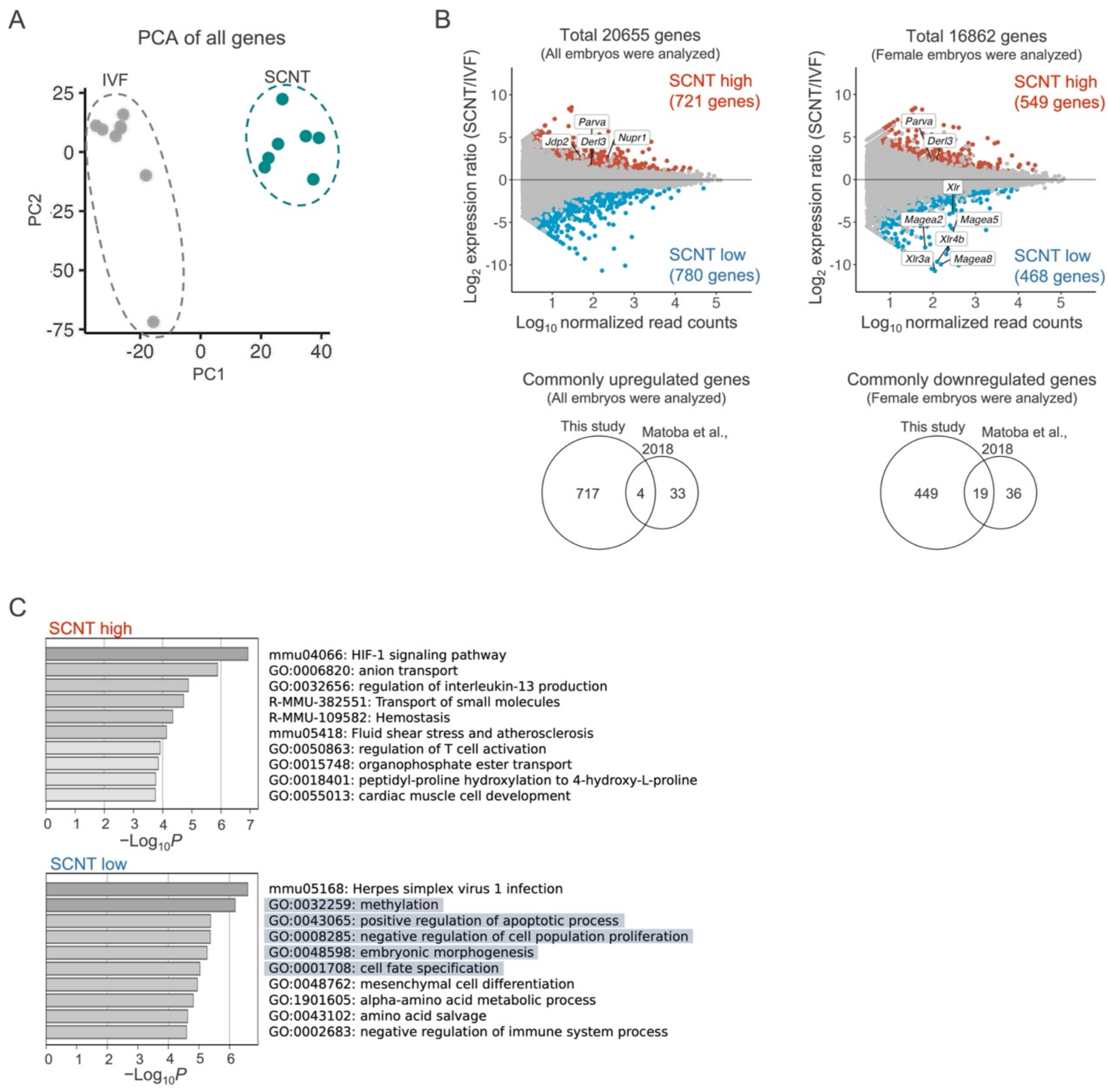
A
PCA of all genes
IVF
SCNT
PC2
PC1
B
Total 20655 genes
(All embryos were analyzed)
SCNT high
(721 genes)
SCNT low
(780 genes)
Parva
Jdp2
Derl3
Nupr1
Log2 expression ratio (SCNT/IVF)
Log10 normalized read counts
Total 16862 genes
(Female embryos were analyzed)
SCNT high
(549 genes)
SCNT low
(468 genes)
Xlr
Magea2
Magea5
Xlr4b
Xlr3a
Magea8
Commonly upregulated genes
(All embryos were analyzed)
This study
Matoba et al., 2018
717
4
33
Commonly downregulated genes
(Female embryos were analyzed)
449
19
36
C
SCNT high
mmu04066: HIF-1 signaling pathway
GO:0006820: anion transport
GO:0032656: regulation of interleukin-13 production
R-MMU-382551: Transport of small molecules
R-MMU-109582: Hemostasis
mmu05418: Fluid shear stress and atherosclerosis
GO:0050863: regulation of T cell activation
GO:0015748: organophosphate ester transport
GO:0018401: peptidyl-proline hydroxylation to 4-hydroxy-L-proline
GO:0055013: cardiac muscle cell development
−Log10P
SCNT low
mmu05168: Herpes simplex virus 1 infection
GO:0032259: methylation
GO:0043065: positive regulation of apoptotic process
GO:0008285: negative regulation of cell population proliferation
GO:0048598: embryonic morphogenesis
GO:0001708: cell fate specification
GO:0048762: mesenchymal cell differentiation
GO:1901605: alpha-amino acid metabolic process
GO:0043102: amino acid salvage
GO:0002683: negative regulation of immune system process
−Log10P

Supplementary Figure S1. Comparison of gene expression between IVF- and SCNT-derived embryos

(A) PCA of the genome-wide gene expression in IVF- (grey) and SCNT-derived (blue) embryos.

(B) (Top left) MA plot showing the gene expression ratios of SCNT- to IVF-derived embryos and the average gene expression of all embryos. Among 20,655 genes, 721 were significantly upregulated (red circles) and 780 were downregulated (blue circles) in SCNT-derived embryos. (Top right) MA plot showing the gene expression ratio of SCNT to female IVF-derived embryos. Among 16,862 genes, 549 were significantly upregulated (red circles) and 468 were downregulated (blue circles) in SCNT-derived embryos. Differentially expressed genes were defined as those with absolute $\log_2$ expression ratio ≥ 1 and adjusted p value < 0.05 using DESeq2. (Bottom left) Venn diagram showing the common SCNT-high genes between this study and an earlier study (Matoba et al., 2018). (Bottom right) Venn diagram showing the common SCNT-low genes between this study and an earlier study (Matoba et al., 2018).

(C) Gene ontology (GO) analysis of SCNT-high (upper) and SCNT-low genes (lower) using Metascape. GO terms related to embryo development are highlighted in blue.

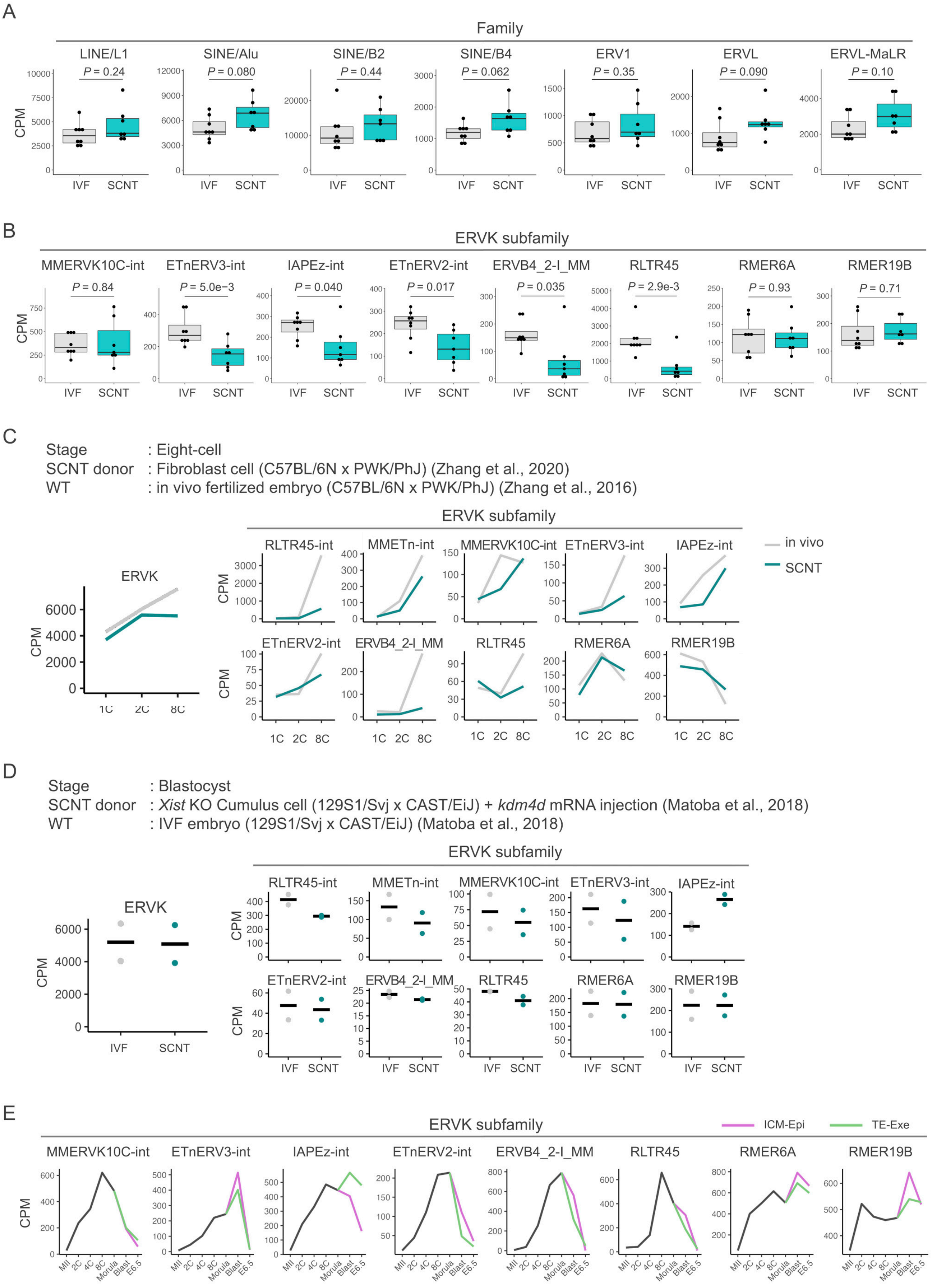
A
Family
LINE/L1
SINE/Alu
SINE/B2
SINE/B4
ERV1
ERVL
ERVL-MaLR
P = 0.24
P = 0.080
P = 0.44
P = 0.062
P = 0.35
P = 0.090
P = 0.10
CPM
IVF
SCNT
B
ERVK subfamily
MMERVK10C-int
ETnERV3-int
IAPEz-int
ETnERV2-int
ERVB4_2-I_MM
RLTR45
RMER6A
RMER19B
P = 0.84
P = 5.0e−3
P = 0.040
P = 0.017
P = 0.035
P = 2.9e−3
P = 0.93
P = 0.71
C
Stage : Eight-cell
SCNT donor : Fibroblast cell (C57BL/6N x PWK/PhJ) (Zhang et al., 2020)
WT : in vivo fertilized embryo (C57BL/6N x PWK/PhJ) (Zhang et al., 2016)
ERVK subfamily
ERVK
RLTR45-int
MMETn-int
MMERVK10C-int
ETnERV3-int
IAPEz-int
ETnERV2-int
ERVB4_2-I_MM
RLTR45
RMER6A
RMER19B
in vivo
SCNT
1C 2C 8C
D
Stage : Blastocyst
SCNT donor : Xist KO Cumulus cell (129S1/Svj x CAST/EiJ) + kdm4d mRNA injection (Matoba et al., 2018)
WT : IVF embryo (129S1/Svj x CAST/EiJ) (Matoba et al., 2018)
ERVK subfamily
ERVK
IVF SCNT
E
ERVK subfamily
ICM-Epi
TE-Exe
MII 2C 4C 8C Morula Blast E6.5

Supplementary Figure S2. Detailed analysis of the expression of TE families and ERVK subfamilies in SCNT-derived embryos

(A) Box-and-whisker plots comparing the expression of TE families between IVF- and SCNT-derived embryos corresponding to Figure 1C (right). *p value* was calculated using a two-tailed *t*-test.

(B) Box-and-whisker plots comparing the expression of ERVK subfamilies covering the top 10 most highly expressed at the morula stage, between IVF- and SCNT-derived embryos corresponding to Figure 1E (right). *p* value was calculated by a two-tailed *t*-test.

(C) Line graph showing transitions in the average expression of ERVK family and its subfamilies in IVF- and SCNT-derived embryos. Published RNA-seq data (GSE71434 and GSE139430) were analyzed. The samples used in the analysis are 1-cell- (1C), 2-cell- (2C), 8-cell- (8C) stage IVF- and SCNT-derived embryos.

(D) Dot plots showing the expression of ERVK family and its subfamilies in IVF- and SCNT-derived embryos. The horizontal lines between dots indicate the mean expression levels. Published RNA-seq data (GSE109214) were analyzed. The samples used in the analysis are IVF and SCNT blastocysts.

(E) Line graph showing the average expression levels of ERVK subfamilies which are the top 10 most highly expressed at the morula stage throughout early embryonic development, corresponding to Figure 1F. Published RNA-seq data (GSE98150) were analyzed.

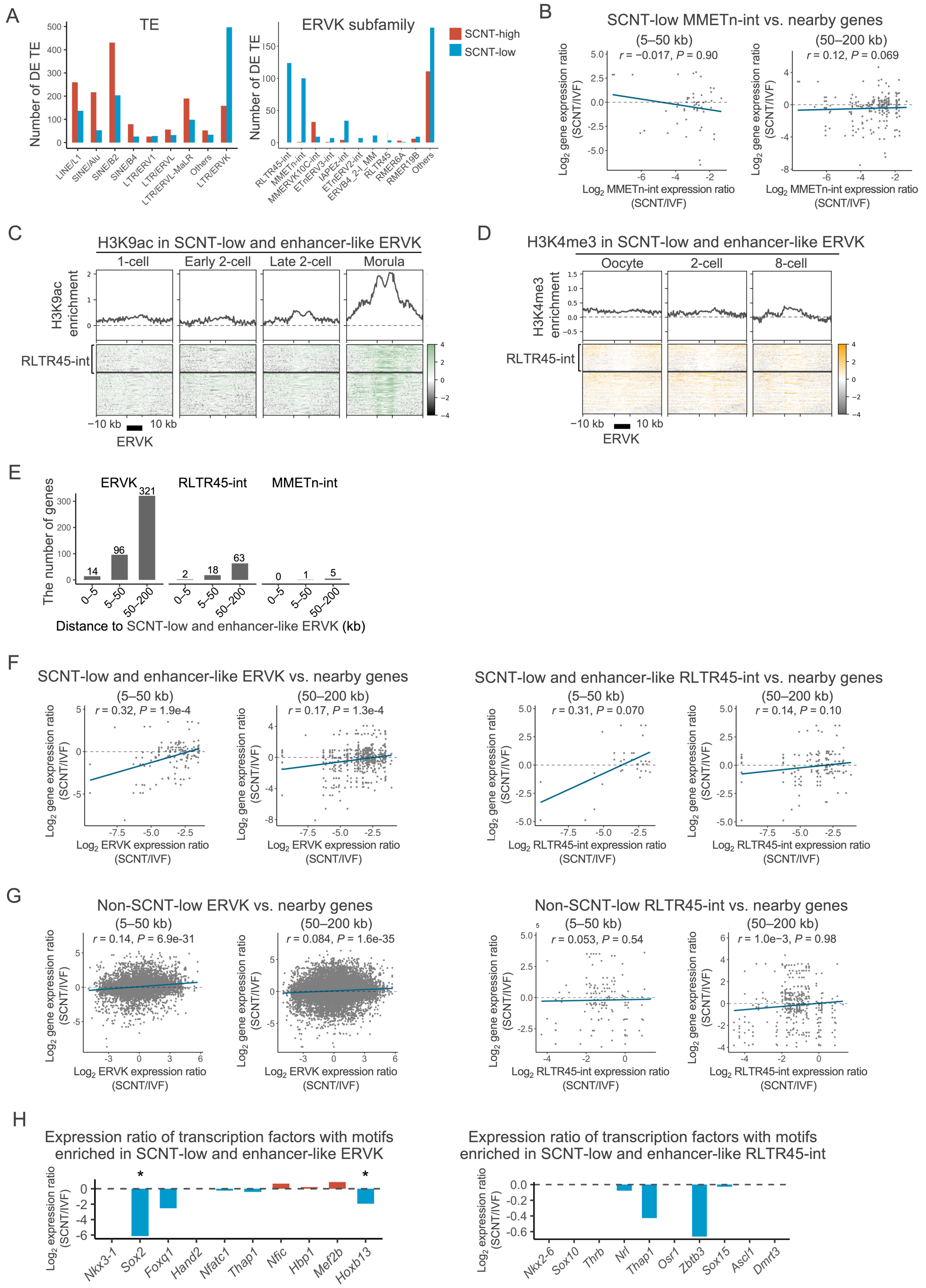
A
TE
ERVK subfamily
SCNT-high
SCNT-low
Number of DE TE
B
SCNT-low MMETn-int vs. nearby genes
(5–50 kb)
r = −0.017, P = 0.90
(50–200 kb)
r = 0.12, P = 0.069
Log2 gene expression ratio (SCNT/IVF)
Log2 MMETn-int expression ratio (SCNT/IVF)
C
H3K9ac in SCNT-low and enhancer-like ERVK
1-cell
Early 2-cell
Late 2-cell
Morula
H3K9ac enrichment
RLTR45-int
−10 kb
10 kb
ERVK
D
H3K4me3 in SCNT-low and enhancer-like ERVK
Oocyte
2-cell
8-cell
H3K4me3 enrichment
RLTR45-int
E
ERVK
RLTR45-int
MMETn-int
The number of genes
Distance to SCNT-low and enhancer-like ERVK (kb)
F
SCNT-low and enhancer-like ERVK vs. nearby genes
(5–50 kb)
r = 0.32, P = 1.9e-4
(50–200 kb)
r = 0.17, P = 1.3e-4
Log2 ERVK expression ratio (SCNT/IVF)
SCNT-low and enhancer-like RLTR45-int vs. nearby genes
(5–50 kb)
r = 0.31, P = 0.070
(50–200 kb)
r = 0.14, P = 0.10
Log2 RLTR45-int expression ratio (SCNT/IVF)
G
Non-SCNT-low ERVK vs. nearby genes
(5–50 kb)
r = 0.14, P = 6.9e-31
(50–200 kb)
r = 0.084, P = 1.6e-35
Non-SCNT-low RLTR45-int vs. nearby genes
(5–50 kb)
r = 0.053, P = 0.54
(50–200 kb)
r = 1.0e−3, P = 0.98
H
Expression ratio of transcription factors with motifs enriched in SCNT-low and enhancer-like ERVK
Log2 expression ratio (SCNT/IVF)
Nkx3-1
Sox2
Foxq1
Hand2
Nfatc1
Thap1
Nfic
Hbp1
Mef2b
Hoxb13
Expression ratio of transcription factors with motifs enriched in SCNT-low and enhancer-like RLTR45-int
Nkx2-6
Sox10
Thrb
Nrl
Thap1
Osr1
Zbtb3
Sox15
Ascl1
Dmrt3

Supplementary Figure S3. Detailed analysis of the expression of TE families and ERVK loci in SCNT-derived embryos

(A) (Left) Bar graphs showing the number of SCNT-high and SCNT-low TE loci at the morula stage. (Right) Bar graphs showing the number of SCNT-high and SCNT-low ERVK subfamily loci. The top 10 most highly expressed and other ERVK subfamilies were analyzed.

(B) Scatter plot showing the Spearman correlation between the expression ratio of SCNT-low ERVK loci (left) or MMETn-int (right) and that of their adjacent genes in SCNT- and IVF-derived embryos, corresponding to Figure 1J.

(C) A heatmap and line graph showing H3K9ac enrichment in SCNT-low, enhancer-like RLTR45-int loci. These show H3K9ac enrichment of 1-cell, early 2-cell, late 2-cell, and morula stage IVF-derived embryos in SCNT-low, enhancer-like ERVK loci. The heatmap is divided into two parts: the upper represents the H3K9ac enrichment of RLTR45-int loci and the lower represents that of the other ERVK loci. Published H3K9ac ChIP-seq data (GSE143523) were analyzed.

(D) A heatmap and line graph showing H3K4me3 enrichment in SCNT-low, enhancer-like RLTR45-int loci among MII oocytes, 2-cell, and 8-cell embryos in SCNT-low, enhancer-like ERVK loci. The heatmap is divided into two parts: the upper represents the H3K4me3 enrichment of RLTR45-int loci and the lower represents that of the other ERVK loci. Published H3K4me3 ChIP-seq data (GSE72784) were analyzed.

(E) Bar graph showing the distribution of genes adjacent to SCNT-low and enhancer-like ERVK, RLTR45-int, and MMETn-int loci.

(F) Scatter plot showing the Spearman correlation between the SCNT/IVF expression ratio of SCNT-low, enhancer-like ERVK (left) or RLTR45-int (right) loci and that of their adjacent genes. Adjacent genes are defined as genes with transcription start site located within 5 to 50 kb or 50 to 200 kb up/downstream from a ERVK locus. The blue line indicates the regression line. *p* values and correlation coefficients were calculated using the 'cor' function in R.

(G) Scatter plot showing the Spearman correlation between the SCNT/IVF expression ratio of non-SCNT-low ERVK (left) or RLTR45-int (right) loci and that of their adjacent genes. Adjacent genes are defined as genes with transcription start site located within 5 to 50 kb or 50 to 200 kb up/downstream from a ERVK locus. The blue line indicates the regression line. *p* values and correlation coefficients were

calculated using the ‘cor’ function in R.

(H) Bar graph showing the $\log_2$ expression ratio of transcription factors with motifs enriched in SCNT-low, enhancer-like ERVK (left) and RLTR45-int (right) loci in IVF and SCNT-derived embryos. Adjusted p values were calculated using DESeq2. * adjusted $p < 0.05$.

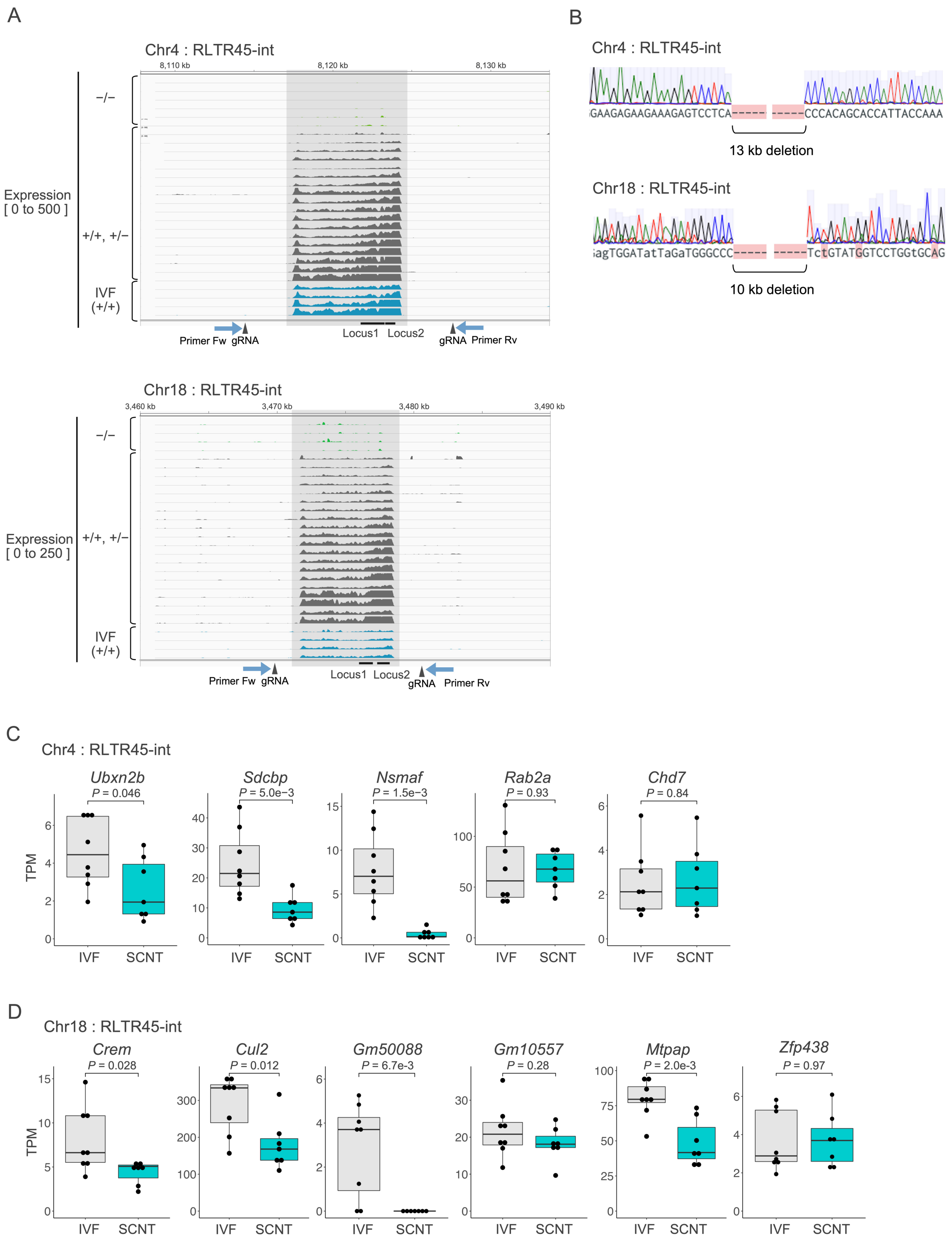
A
Chr4 : RLTR45-int
8,110 kb
8,120 kb
8,130 kb
−/−
Expression
[0 to 500]
+/+, +/−
IVF
(+/+)
Primer Fw gRNA
Locus1 Locus2
gRNA Primer Rv
Chr18 : RLTR45-int
3,460 kb
3,470 kb
3,480 kb
3,490 kb
−/−
Expression
[0 to 250]
+/+, +/−
IVF
(+/+)
Primer Fw gRNA
Locus1 Locus2
gRNA Primer Rv
B
Chr4 : RLTR45-int
GAAGAGAAGAAAGAGTCCTCA
CCCACAGCACCATTACCAAA
13 kb deletion
Chr18 : RLTR45-int
agTGGATatTaGaTGGGCCC
TctGTATGGTCCTGGtGCAG
10 kb deletion
C
Chr4 : RLTR45-int
Ubxn2b
P = 0.046
Sdcbp
P = 5.0e−3
Nsmaf
P = 1.5e−3
Rab2a
P = 0.93
Chd7
P = 0.84
TPM
IVF
SCNT
D
Chr18 : RLTR45-int
Crem
P = 0.028
Cul2
P = 0.012
Gm50088
P = 6.7e-3
Gm10557
P = 0.28
Mtpap
P = 2.0e-3
Zfp438
P = 0.97
TPM
IVF
SCNT

Supplementary Figure S4. Expression analysis of genes around knockout target regions in IVF- and SCNT-derived embryos

(A) Genome browser view showing the expression levels in putatively homozygous knockout ($^{-/-}$) embryos and other embryos ($^{+/+}$, $^{+/-}$), together with those in representative non-treated IVF embryos ($^{+/+}$). The RLTR45-int regions on chromosome 4 (top) and 18 (bottom) removed by the gRNAs-Cas9 complexes are highlighted in gray. Arrow heads show the gRNA-targeting regions.

(B) Representative view of deleted regions on chromosome 4 (top) and 18 (bottom) validated by Sanger sequencing. Sanger sequencing was performed by using PCR products amplified around the deleted regions from DNA extracts of mouse tails.

(C) Box-and-whisker plots comparing the expression levels of the nearby genes of RLTR45-int on chromosome 4 between IVF- and SCNT-derived embryos. p values were calculated by a two-tailed t-test.

(D) Box-and-whisker plots comparing the expression levels of the nearby genes of RLTR45-int on chromosome 18 between IVF- and SCNT-derived embryos. p values were calculated by a two-tailed t-test.

Supplementary Table S1. Sequence information of the primers used for genotyping or the synthesis of sgRNAs.

Primer/sgRNA name	Application	Fw or Rv	Sequence
sgRNA_RLTR45-int.on.chr4_5'cut	sgRNA synthesis	Fw	TAATACGACTCACTATAGAGTCCATTAAAAGACTCGGG
		Rv	TTCTAGCTCTAAAACCCCGAGTCTTTTAATGGACT
sgRNA_RLTR45-int.on.chr4_3'cut	sgRNA synthesis	Fw	TAATACGACTCACTATAGGAAGCGTATGAAGCAGACGT
		Rv	TTCTAGCTCTAAAACACGTCTGCTTCATACGCTTC
sgRNA_RLTR45-int.on.chr18_5'cut	sgRNA synthesis	Fw	TAATACGACTCACTATAGGATATTAGATGGGCCCCTAG
		Rv	TTCTAGCTCTAAAACCTAGGGGCCCATCTAATATC
sgRNA_RLTR45-int.on.chr18_3'cut	sgRNA synthesis	Fw	TAATACGACTCACTATAGCGGTATGGTCCTGATCGGTA
		Rv	TTCTAGCTCTAAAACTACCGATCAGGACCATACCG
Primer_RLTR45-int.on.chr4_outside	genotyping	Fw	TACTGGAGAGAAAGAGGTCACCACAGAG
		Rv	CGGCTGTACCTCTCAGGATATATAGGCA
Primer_RLTR45-int.on.chr4_inside	genotyping	Fw	TTTGATCCAGCAGCCTACGTT
		Rv	ATCAGGACTTGCTGGGCTTAC
Primer_RLTR45-int.on.chr18_outside	genotyping	Fw	CTATAGAAAGGGTTTCAGGCCAGCCAAA
		Rv	GACAATAACCCAACAATGCAGAAGCCTG
Primer_RLTR45-int.on.chr18_inside	genotyping	Fw	TGCTCCATTGTGAACCCCTC
		Rv	TCAGGAAAACCCACCTTCGG
